# Meningeal macrophages regulate fibroblasts to influence meningeal lymphatic function following traumatic brain injury

**DOI:** 10.7150/thno.130263

**Published:** 2026-03-04

**Authors:** Xiaoming Guo, Yueli Zhu, Shiqi Gao, Yiwen Wu, Yuan Hong

**Affiliations:** 1 Department of Neurosurgery, The Second Affiliated Hospital, Zhejiang University School of Medicine, Hangzhou, China.; 2 Clinical Research Center for Neurological Diseases of Zhejiang Province, Hangzhou, China.; 3 Key Laboratory of Precise Treatment and Clinical Translational Research of Neurological Diseases, Hangzhou, China.; 4 Department of Geriatrics, The First Affiliated Hospital, Zhejiang University School of Medicine, Hangzhou, China.

**Keywords:** fibroblast, macrophage, meningeal lymphatic vessel, traumatic brain injury, VEGF-C

## Abstract

**Background:**

The meningeal lymphatic system represents a critical pathway for cerebrospinal fluid exchange, waste clearance, and immune cell trafficking in the central nervous system. Although traumatic brain injury (TBI) induces marked alterations in meningeal immune cells, their role in regulating meningeal lymphatic function under physiological and pathological conditions remains unclear.

**Methods:**

Single-cell RNA sequencing, confocal microscopy, and flow cytometry were performed to identify a distinct population of meningeal fibroblasts that secrete VEGF-C and to investigate alterations in this population following TBI. By subdural injection of clodronate liposomes and PDGF-C, the role of meningeal resident macrophages and their secreted PDGF-C in regulating meningeal lymphatic function was further examined.

**Results:**

A distinct population of meningeal fibroblasts was identified as a source of VEGF-C, whose production is regulated by meningeal macrophages through the PDGF-C/PDGFRα axis. In the early phase of TBI, depletion of resident meningeal macrophages impaired fibroblast-derived VEGF-C production. Further investigations revealed that macrophage depletion resulted in severe meningeal lymphatic dysfunction. Conversely, subdural administration of PDGF-C after TBI enhanced fibroblast-derived VEGF-C production, restored meningeal lymphatic function, alleviated neuroinflammation, and promoted myelin integrity.

**Conclusion:**

Overall, our findings emphasize that meningeal resident macrophages can regulate fibroblast-derived VEGF-C production via PDGF-C, thereby influencing meningeal lymphatic function, and thus propose a novel therapeutic strategy to enhance neurological repair after TBI.

## Introduction

The central nervous system (CNS) has traditionally been considered an immunologically privileged organ, protected behind the blood-brain barrier and thought to lack a lymphatic system. However, the recent discovery of meningeal lymphatic vessels (mLVs) challenged this view and offered a new perspective on the immune system in the CNS. A growing body of evidence indicates that brain borders (including meninges, perivascular spaces and choroid plexus) are pivotal sites for the active exchange of immune signals between the CNS and the peripheral immune system 1,2. Based on this, the meninges serve as both a physical barrier and a dynamic immune surveillance hub. Via the meningeal lymphatic network, immune cells, cytokines, and antigens can be drained and redistributed 3. However, this homeostatic immune interface is altered under pathological conditions. Besides, the discovery of mLVs in the past decade has revealed a critical link between impaired brain waste clearance and the processes of aging and neurodegenerative diseases.

Traumatic brain injury (TBI), a leading global cause of death and disability, affects millions of people annually worldwide 4,5. It has been reported that TBI causes long-lasting deficits in meningeal lymphatic drainage and eventual neurological dysfunction 6,7. In the acute phase post-injury, elevated intracranial pressure is likely a major factor contributing to impaired meningeal lymphatic function, as it directly disrupts cerebrospinal fluid (CSF) and interstitial fluid drainage. As the disease progresses, delayed growth of mLVs may represent a compensatory adaptation to restore CSF dynamics and waste clearance 6. Therefore, promoting the proliferation of mLVs may offer a promising therapeutic approach for acute TBI by restoring mLV drainage function, alleviating neuroinflammation driven by damage-associated molecular patterns, and ultimately facilitating neurological recovery. To date, accumulating evidence has highlighted the crucial supportive and regulatory roles of VEGF-C in meningeal lymphatic function. Research has demonstrated that in pup mice, paracrine VEGF-C drives the sprouting of VEGFR3-positive lymphatic endothelial cell (LEC) progenitors from the common cardinal vein, leading to the formation of the first lymphatic plexus 8,9. In adult mice, targeting VEGFR3 via genetic deletion or pharmacological blockade effectively disrupts VEGF-C/VEGFR3 signaling and induces regression of mLVs 9,10. The above findings support the hypothesis that the meninges contain sustained VEGF-C-secreting cells. Several studies have reported that PDGFRα^+^ fibroblasts and radial astrocytes may constitute cellular sources of VEGF-C in the zebrafish model 11,12. Despite these findings, the precise cellular sources of VEGF-C in mammalian meninges have not yet been systematically investigated. Building on the above findings, we posited that VEGF-C may be produced by fibroblasts.

The interaction between immune cells and mLVs has emerged as a research hotspot in neuroimmunology. Although immune cells (except microglia) are excluded from brain parenchyma under physiological conditions, the meninges harbor a diverse array of immune cells 13,14. Recent studies have demonstrated the impact of mLVs on myeloid cell and T cell infiltration in the brain 2,14. However, it remains unknown whether the immune cells can, in turn, regulate mLVs. Meningeal macrophages are reported to play a role in monitoring and filtering the CSF, facilitating the scavenging of antigens and metabolites derived from it 15. Besides, a prior study demonstrated an association between spinal cord macrophages and meningeal LECs in lymphangiogenic signaling after spinal cord injury, as revealed by transcriptomic analysis, indicating a potential close interplay between meningeal macrophages and the regulation of meningeal lymphatic drainage 16. However, the precise mechanism has not yet been elucidated. CNS fibroblasts, enriched at CNS borders, play key roles in maintaining meningeal and vascular structure, promoting immune surveillance, and potentially regulating CSF-interstitial fluid exchange 17. Many existing studies have emphasized the reciprocal interactions between fibroblasts and macrophages under different disease conditions 18-20. A recent study demonstrated that interactions between fibroblasts and immune cells shape recovery after brain injury, and ligand-receptor analysis highlighted *Tgfb1* as a macrophage ligand that could support myofibroblasts 21. However, it remains unclear whether macrophages and fibroblasts engage in direct communication and how they might influence mLv function after acute TBI.

In this study, we performed single-cell RNA sequencing (scRNA-seq), laser confocal microscopy, and flow cytometry to identify a distinct population of meningeal fibroblasts secreting VEGF-C. Our findings revealed that depletion of resident meningeal macrophages impairs meningeal fibroblast ability to produce VEGF-C in the early phase of TBI and identified PDGF-C as a key regulator of meningeal fibroblast function. Notably, subdural administration of PDGF-C can significantly enhance meningeal lymphatic drainage, improve white matter integrity, and alleviate neuroinflammation following TBI. Thus, our study highlights the effects of meningeal macrophages on fibroblasts and suggests a novel therapeutic intervention to promote neurological repair after TBI.

## Methods

### Animal

Young (8-10-week-old) male C57BL/6J mice were obtained from SLAC Laboratory Animal Company (Shanghai, China). The mice were housed in a controlled environment (22 ± 2 °C, 50-60% humidity, 12-h light/dark cycle), with unrestricted access to food and water. Following a 1-week acclimation period, all mice were randomly allocated to either closed-head TBI modeling or other treatments using a computer-generated randomization sequence (Microsoft Excel RAND function).

### Closed-head TBI modeling

The closed-head TBI model in mice was adapted from a previously reported method 22. After anesthesia and analgesic treatment, mice were placed on a stereotaxic frame, and the head was maintained using both nose and ear bars. To minimize injury to the blood vessels and lymphatic vessels, the TBI model was performed over the right parietal bone (centered 2 mm anterior and 3.5 mm lateral to bregma). We used a 3-mm rigid impactor driven by an electromagnetic-controlled impact device (PinPoint PCI3000, USA) to establish the TBI model (velocity: 3 m/s, depth: 2.2 mm, duration: 200 ms). Animals in the sham group were treated identically, except that no impact was applied. All mice were postoperatively maintained on a heating pad until regaining consciousness and were subsequently administered analgesics.

### Subdural injection and subdural trocar system

The experimental animal was secured in a stereotactic frame (RWD Life Science, China) with its head slightly tilted to the right, lowering the right hemisphere. Under a surgical microscope, a site close to the superior sagittal sinus but away from major vessels was selected. A small hole was carefully drilled vertically through the skull using a sharp 31-G needle, and the dura was carefully pierced while avoiding separation from the skull. Subsequently, the needle was replaced with a 31-G blunt needle attached to a microsyringe for injection. For clodronate liposomes (CLO) or PBS liposomes (5 mg/mL, 40337ES08, 40338ES08, Yeasen) delivery experiments, 10 µL of liposomes were injected into the subdural space at a rate of 5 µL/min. For subdural injection of the fluorescent tracers, namely OVA (45 kDa, 1 mg/mL diluted in artificial CSF, O34784, Invitrogen) and beads (1µm, F8823, Invitrogen), a dose of 5 µL was injected at a rate of 5 µL/min.

For AAV delivery experiments, 5 µL of artificial CSF containing 5 × 10¹² genome copies/mL of AAV9-mCOL1A2-PDGFRα or AAV9-Control (purchased from GeneChem, Shanghai, China) was injected into the subdural space at a rate of 5 µL/min. After completion of the infusion, the needle was kept in place for 3 min before withdrawal, and the injection site was sealed with sterile bone wax.

The subdural trocar system (RWD Life Science, China) was implanted following the standard procedure for subdural injection. The main difference was that a specially designed, graduated cannula needle was inserted into the skull and secured at its proximal end to the skull surface with an adhesive base to prevent displacement. An external injection needle was then repeatedly inserted through the cannula into the subdural space, allowing multiple administrations of PDGF-C (0.1 μg/μL, HY-P73354, MCE) over several days while minimizing tissue damage from repeated operations. This system was firmly attached to the base, enabling the mice to move freely.

### Intra-cisterna magna (i.c.m.) injection

The mouse was secured in a stereotaxic frame, and a midline incision was made over the occipital region. The neck muscles were retracted, leaving a thin layer of deep neck muscles over the dura when the cisterna magna was exposed to prevent backflow upon needle withdrawal. Then, 2 µL of FluoSpheres carboxylate beads (0.5 µm, F8813, Invitrogen) were injected at 2 µL/min using the microsyringes. At the end of the injection, the needle was left in place for an additional 3 min. The cervical skin was then sutured, and the mouse was allowed to recover on a heating pad. One hour later, the mouse was euthanized for tissue collection.

### Tissue collection and processing

Under deep anesthesia, brain and meningeal tissues were collected from mice following transcardial perfusion with ice-cold PBS. Meningeal tissues were microdissected in ice-cold DMEM and enzymatically digested at 37 °C for 30 min with Liberase TM (4 µg/mL, 5401119001, Roche) and DNase I (50 U/mL, 10104159001, Roche), with inversion and gentle shaking every 5 min. Digestion was terminated by adding 2 volumes of ice-cold PBS, and the suspension was filtered through a 70-µm cell strainer, then centrifuged at 400 g for 5 min at 4 °C. The cell pellet was then resuspended in FACS buffer for subsequent scRNA-seq and flow cytometry.

The right hemispheres were isolated after brain dissection, and the tissues were dissociated into homogenates using the Neural Tissue Dissociation Kit (T) in combination with a gentleMACS™ Octo Dissociator with Heaters (Miltenyi Biotec), according to the manufacturer's protocol. The homogenates were passed through a 70-µm cell strainer, mixed with 30% Percoll (17089109, Cytiva), and subjected to density gradient centrifugation to remove myelin debris. The cell pellet was then resuspended in FACS buffer for subsequent flow cytometry.

### Flow cytometry analysis

Red blood cells were lysed using 1× RBC Lysis Buffer (00-4333-57, Thermo Fisher Scientific). The resulting suspensions were incubated with CD16/32 antibody (156603, BioLegend) to block Fc receptors. Cell viability was assessed by staining with Zombie NIR (423106, BioLegend) for 15 min, followed by surface antibody staining in the dark for 30 min: anti-CD163-Super Bright 702 (67-1631-82, Thermo Fisher Scientific), anti-CD31-APC (102509, Biolegend), anti-CD45-BV605 (103155, Biolegend), anti-CCR2-BV650 (150613, Biolegend), anti-PDGFRα-PerCP/Cy5.5 (135914, Biolegend), anti-I-A/I-E-PE (107607, Biolegend), anti-CD4-BV785 (100551, Biolegend), anti-CD3e-FITC (11-0031-81, Thermo Fisher Scientific), anti-CD19-PE (561736, BD Biosciences), anti-CD11b-PE-CY7 (25-0112-81, Thermo Fisher Scientific), anti-CD11c-BV510 (117337, Biolegend), anti-CD8a-PercP/Cy5.5 (100733, Biolegend), and anti-Ly6G-Pacific Bule (127611, Biolegend). For intracellular detection of CD206 (141717, Biolegend) and VEGF-C (sc-374628 AF488, Santa Cruz), cells were fixed and permeabilized with the Fixation/Permeabilization Kit (554714, BD Biosciences, USA), then incubated with the respective antibodies for 30 min. Flow cytometric analysis was performed on a BD LSRFortessa™ (BD Biosciences), and data were processed using FlowJo software (Tree Star, Ashland).

### Deep cervical lymph node (dCLN) clearance

dCLN clearance was performed according to the published CUBIC protocol and our published study with minor modifications 23,24. Briefly, nodes were sequentially incubated in 50% Reagent 1 (1:1 in dH₂O) and Reagent 1 for 1 day each at 37 °C with shaking, supplemented with DAPI (1:1000). After washing twice in PBS containing 0.01% sodium azide (2 h and overnight), nodes were incubated in 50% Reagent 2 (1:1 in dH₂O) for 1 day at 37 °C with DAPI, followed by Reagent 2 for another day under the same conditions. Finally, nodes were mounted in eight-well chambers (155411, Thermo Fisher Scientific) with mineral oil and imaged using confocal microscopy.

### Immunohistochemistry and image analysis

After perfusion, mouse brains were post-fixed in 4% PFA at 4 °C overnight, followed by cryoprotection in graded sucrose solutions (15% and 30%). Samples were embedded in optimal cutting temperature compound, stored at -80 °C, and sectioned at 25 µm thickness as needed for staining. For meningeal staining, skulls with attached meninges were fixed in 4% PFA for 48 h. Under a microscope, the meninges were carefully dissected intact, then blocked and permeabilized with QuickBlock™ Blocking Buffer (P0260, Beyotime) at 4 °C overnight. Samples were then incubated with primary antibodies overnight at 4 °C, followed by incubation with the corresponding fluorescently labeled secondary antibodies (1:500, Alexa Fluor, Invitrogen). Between each step, samples were thoroughly washed with 0.01 M PBS. Finally, samples were mounted with DAPI-containing antifade mounting medium (ab104139, Abcam) and imaged with a Leica DMi8 confocal microscope. The primary antibodies used for immunohistochemistry included: Rat Anti-CD31 (1:100, sc-18916, Santa Cruz), Rabbit Anti-VEGF-C (1:200, ab9546, Abcam), Rat Anti-PDGFRα (1:200, 14-1401-82, Thermo Fisher Scientific), Goat anti-CD206 (1:200, AF2535, R&D Systems), Rat anti-LYVE-1 (1:100, 14-0443-82, Thermo Fisher Scientific), Goat anti-LYVE-1 (1:200, AF2125, R&D Systems), Rat anti-CD163 (1:200, 14-1631-82, Thermo Fisher Scientific), Rabbit Anti-CCR2 (1:200, ab273050, Abcam), Rabbit Anti-CD68 (1:200, PA578996, Thermo Fisher Scientific), Goat anti-IBA-1 (1:200, ab5076, Abcam), Rabbit anti-IBA-1 (1:500, 019-19741, Wako), Rat anti-MBP (1:200, ab7349, Abcam), Mouse anti-SMI32 (1:200, 801701, BioLegend), Rabbit Anti-GFAP (1:200, 80788, CST), and Goat anti-EphB4 (1:200, AF446, R&D Systems).

For quantitative analysis of VEGF-C⁺ cells, LYVE-1 coverage, and bead coverage in the meninges, consistent region of interest (ROI) selection criteria were applied. For the quantitative analysis of CD68/IBA-1 and SMI/MBP, corresponding serial sections were selected to ensure ROI consistency. The recorded images were manually quantified using ImageJ by two independent observers blinded to the group allocation.

### scRNA-seq and analysis

In brief, single-cell suspensions were loaded onto Chromium microfluidic chips with 3′ chemistry and processed using a 10× Chromium Controller (10X Genomics) to introduce cell-specific barcodes. The captured RNA was reverse-transcribed, and sequencing libraries were constructed using the Chromium Single Cell 3′ reagent kit (10X Genomics) according to the manufacturer's protocol. Libraries were sequenced on an Illumina NovaSeq 6000 platform. Raw scRNA-seq data were initially processed and visualized using Seurat (v5.2.1) in R (v4.4.3). Cell-cell communication analysis was performed with Cellchat (v1.6.1), and the activity of selected gene sets was quantified with AUCell (v1.25.2). The functional gene sets were collected based on Gene Ontology (GO) terms (Table S1).

### Quantitative polymerase chain reaction (qPCR)

The mRNA was extracted from the harvested meninges using TRIzol reagent (15596018, Thermo Fisher Scientific). Subsequently, cDNA was synthesized from mRNA using a reverse transcription kit (R323, Vazyme). Ct values were first normalized to the endogenous control gene GAPDH to obtain ΔCt. The ΔΔCt value was then determined as the difference between the ΔCt of the post-TBI time points and the respective value from the sham group. Relative gene expression levels were expressed using the formula: 2^-ΔΔCt^. The primer sequences used are listed in Table S2.

### Enzyme-linked immunosorbent assay (ELISA)

Protein was extracted from fresh meninges and quantified using an ELISA kit (A104838, Shanghai Fusheng) according to the manufacturer's instructions. Briefly, protein supernatant was applied to ELISA plates. After incubation with specific antibodies and the substrate, the optical density was measured to determine protein concentration.

### Behavioral tests

A series of behavioral tests with high accuracy and sensitivity in evaluating subacute sensorimotor deficits and chronic memory impairments in rodents was conducted 25,26. The experimenters were blinded to treatment groups during all behavioral testing.

### Adhesive removal test

To evaluate forepaw sensitivity and motor function, a small piece of adhesive tape (0.1 cm²) was attached to the left forepaw (contralateral to the affected hemisphere), and the time taken by the mice to completely remove the tape was recorded, with a maximum of 1 min. Mice were pre-trained for three consecutive days prior to TBI induction. On the testing day, three trials were performed per mouse, and the average latency was calculated for analysis.

### Cylinder test

Mice were placed in a transparent cylinder (10 cm diameter × 17.5 cm height), and their spontaneous exploration, rearing, and wall contacts were recorded for 5 min using a video camera to assess forelimb asymmetry. During vertical exploration, wall contacts were categorized based on the forelimb(s) employed: ipsilateral (left), contralateral (right), or simultaneous use of both forelimbs. Videos were analyzed by an independent observer blinded to the experimental groups, and the asymmetry rate was calculated as: (R-L)/(L+R+B) × 100%.

### Foot fault test

Mice were placed on an elevated grid surface, and their movements were video recorded for 2.5 min. A foot fault was defined as a slip of the impaired forepaw (left, contralateral to the affected hemisphere). All videos were subsequently analyzed by an investigator blinded to grouping, and the percentage of foot faults relative to total steps was calculated.

### Morris water maze test

Cognitive performance was assessed using the Morris water maze test, as previously described 27. The apparatus consisted of a circular pool (diameter 120 cm, height 50 cm) filled with opaque water and divided into four quadrants. A circular platform was positioned 1 cm below the water surface in the first quadrant. All mice received four training sessions per day for four consecutive days (24-27 days post-TBI), with 60 s to find the hidden platform. For each daily trial, mice were randomly placed into the water at one of the four starting locations. Swimming trajectories were recorded using an automated behavioral analysis system. The escape latency in each trial, defined as the time to find the hidden platform, was used as an index of spatial learning. Any subject failing to find the platform within the 60-second cutoff was guided to it and permitted to remain there for a 15-second consolidation period. On day 5, mice were released from the pool wall in the third quadrant (the location farthest from the platform) and allowed to swim freely for 60 s. The time spent in the target quadrant and the number of crossing the platform were recorded.

### Novel Y maze

Cognitive function was further assessed using a modified Y-maze test 28. The apparatus consisted of three identical arms arranged at 120° angles. During the training phase, the entrance to the novel arm was blocked, allowing mice to freely explore only the start arm and the other familiar arm for 10 min for habituation. In the subsequent test phase, the barrier was removed to allow free access to all three arms. Mice with intact cognitive function typically exhibit an innate preference for novelty, manifested by significantly increased time spent exploring the novel arm. All sessions were recorded using an automated video-tracking system, and the time spent in the novel arm was quantified for analysis.

### Novel object recognition test

On day 1, mice were allowed to freely explore an open-field box (35 cm × 35 cm × 40 cm) for 10 min. On day 2, two identical objects were placed on opposite sides of the box, and mice were allowed to explore them for 10 min. After 24 h, one of the objects was replaced with a novel one, with a different color and shape. The mice were allowed to explore for 5 min. The recognition index was calculated as the percentage of time spent exploring the novel object relative to the total time spent exploring both objects.

### Statistical analysis

Results were presented as mean ± standard deviation. For normally distributed continuous variables, comparisons between two groups were performed using Student's t-test, while comparisons among three or more groups were analyzed by one-way ANOVA with Bonferroni's post-hoc test. For non-normally distributed variables, the Mann-Whitney test (two groups) or Kruskal-Wallis test (three or more groups) was applied, followed by Dunn's post-hoc test. Differences in means across groups with repeated measures over time were analyzed using a two-way repeated-measures ANOVA. A *P* value less than 0.05 was considered statistically significant, and *P* values were indicated on the respective graphs whenever possible. All statistical analyses were performed using GraphPad Prism software (version 8.0.2).

## Results

### Presence of VEGF-C^+^ fibroblasts in the meninges and their acute post-traumatic depletion

**Figure 1:**
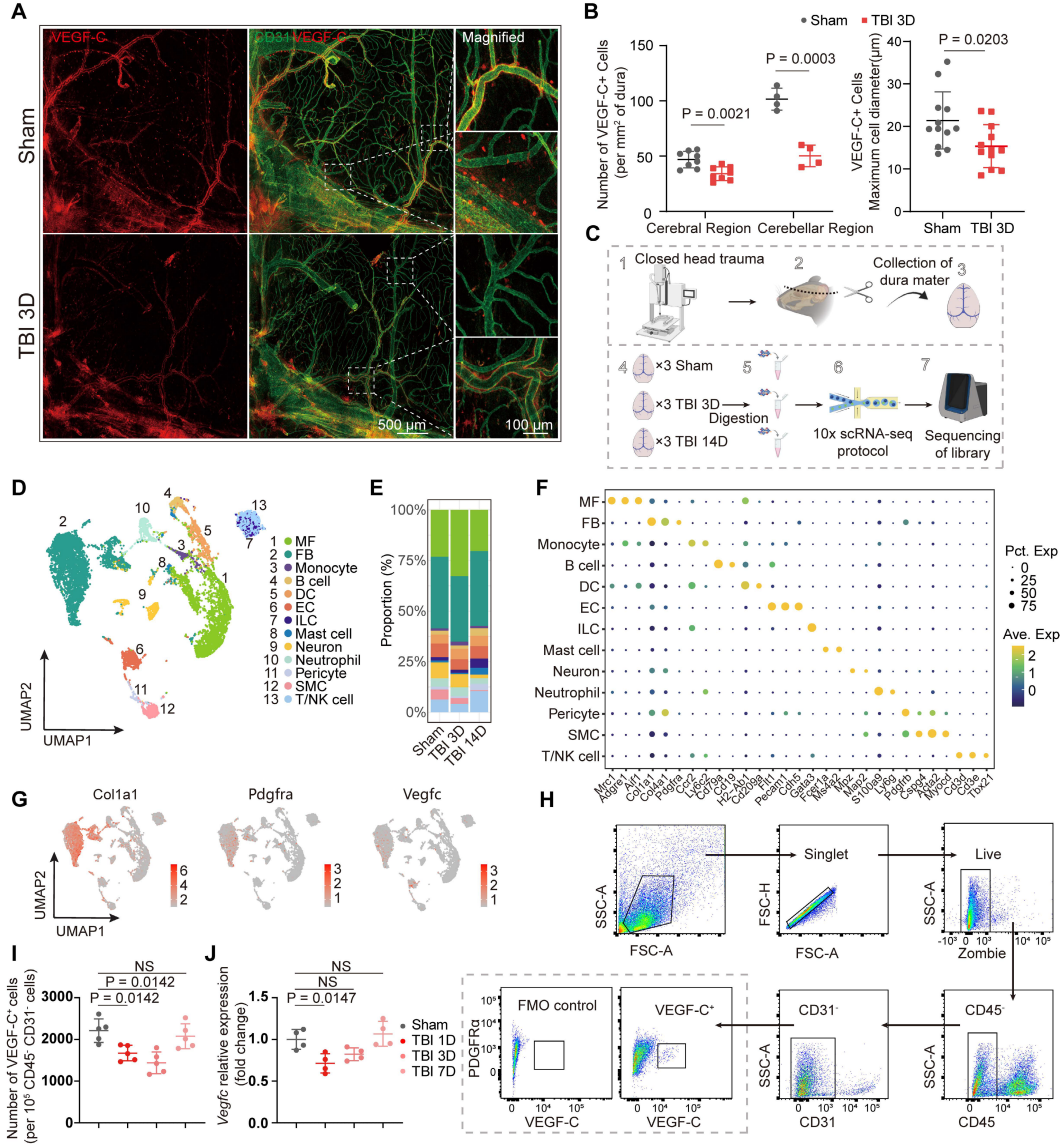
**Presence of VEGF-C^+^ fibroblasts in the meninges and their acute post-traumatic depletion. (**A) Representative images of meninges isolated from sham and 3 days post-TBI groups, staining for VEGF-C (red) and CD31 (green). (B) Quantification of VEGF-C⁺ cell numbers and maximal diameters in cerebral and cerebellar regions of mice at 3 days post-TBI versus the sham group (n = 4 mice). (C) Study design for scRNA-seq and validation experiments. Each scRNA-seq sample was derived from 3 mice. (D) Uniform manifold approximation and projection (UMAP) visualization of 13 clusters with corresponding annotations. (E) Stacked bar plot showing the relative proportions of each cell cluster across groups. (F) Dot plots showing characteristic marker genes with corresponding average expression (color) and proportion of expressing cells (dot size) across cell types. (G) UMAP reflecting expression levels of* Col1a1, Pdgfra, and Vegfc*. (H, I) Flow cytometric quantification of CD45⁻CD31⁻VEGF-C⁺ meningeal cells in TBI and sham mice (normalized per 10⁵ CD45⁻CD31⁻ cells, n = 5). (J) Relative mRNA expression of *Vegfc* in the meninges of sham and TBI mice (n = 4). MF: macrophage, FB: fibroblast, DC: dendritic cell, EC: endothelial cell, ILC: innate lymphoid cell, SMC: smooth muscle cell.

Given that sustained VEGF-C support is required for the maintenance of mLV morphology and function, we hypothesized that the meninges contain abundant VEGF-C-secreting cells 9,10. Herein, we identified abundant VEGF-C⁺ cells in the mouse meninges, distributed homogeneously around meningeal blood vessels (Figure 1A, Figure S1A). Besides, a subset of cells with lower VEGF-C expression spatially overlapped with vascular regions. Notably, the abundance of VEGF-C⁺ cells in the meninges decreased after closed-head TBI compared with the sham group, and the cell diameter was also reduced (Figure 1A-B, Figure S1B).

To comprehensively characterize the properties of these cells and substantiate the alterations observed following TBI, we performed scRNA-seq of the meninges from three experimental groups: sham controls and mice at 3 and 14 days post-TBI. Assessments at 3 and 14 days post-TBI corresponded to the subacute and chronic recovery phases. The closed-head injury model was utilized to prevent confounding meningeal damage and aligned with established methodological standards. Moreover, this model can result in extensive injury to the cortex, axons, and white matter nuclei, which better reflects clinical reality. Due to the small volume of each meningeal sample, we pooled meninges from three animals per group for sequencing (Figure 1D). After quality control and correction for batch effects, a total of 6493, 4379, and 4233 cells from the sham group and mice at 3 days and 14 days post-TBI, respectively, were included for downstream analyses. Unsupervised clustering revealed 13 distinct clusters, and subsequent annotation was based on their gene expression profiles (Figure 1D-F). Importantly, meningeal fibroblasts and macrophages constituted the major cellular components of the meninges (Figure 1E). To confirm the identity of VEGF-C⁺ cells, we examined the expression of marker genes, including *Col1a1*, *Pdgfra*, and *Vegfc*, and established fibroblasts as the predominant source of VEGF-C (Figure 1G, Figure S1C). After identifying these VEGF-C-positive cells as putative fibroblasts, flow cytometry was used to examine the temporal dynamics of CD45⁻CD31⁻VEGF-C⁺ fibroblast populations in the meninges at multiple time points (0, 1, 3, and 7 days) after TBI (Figure 1H-I, Figure S1D). We found that the abundance of VEGF-C⁺ cells was significantly reduced at 1 and 3 days after TBI compared with the sham group but returned to initial levels at 7 days after TBI (Figure 1I). In addition, transcriptional changes in *Vegfc* in the meninges after TBI were validated by qPCR, which showed *Vegfc* downregulation in the acute phase of TBI (Figure 1J).

**Figure 2:**
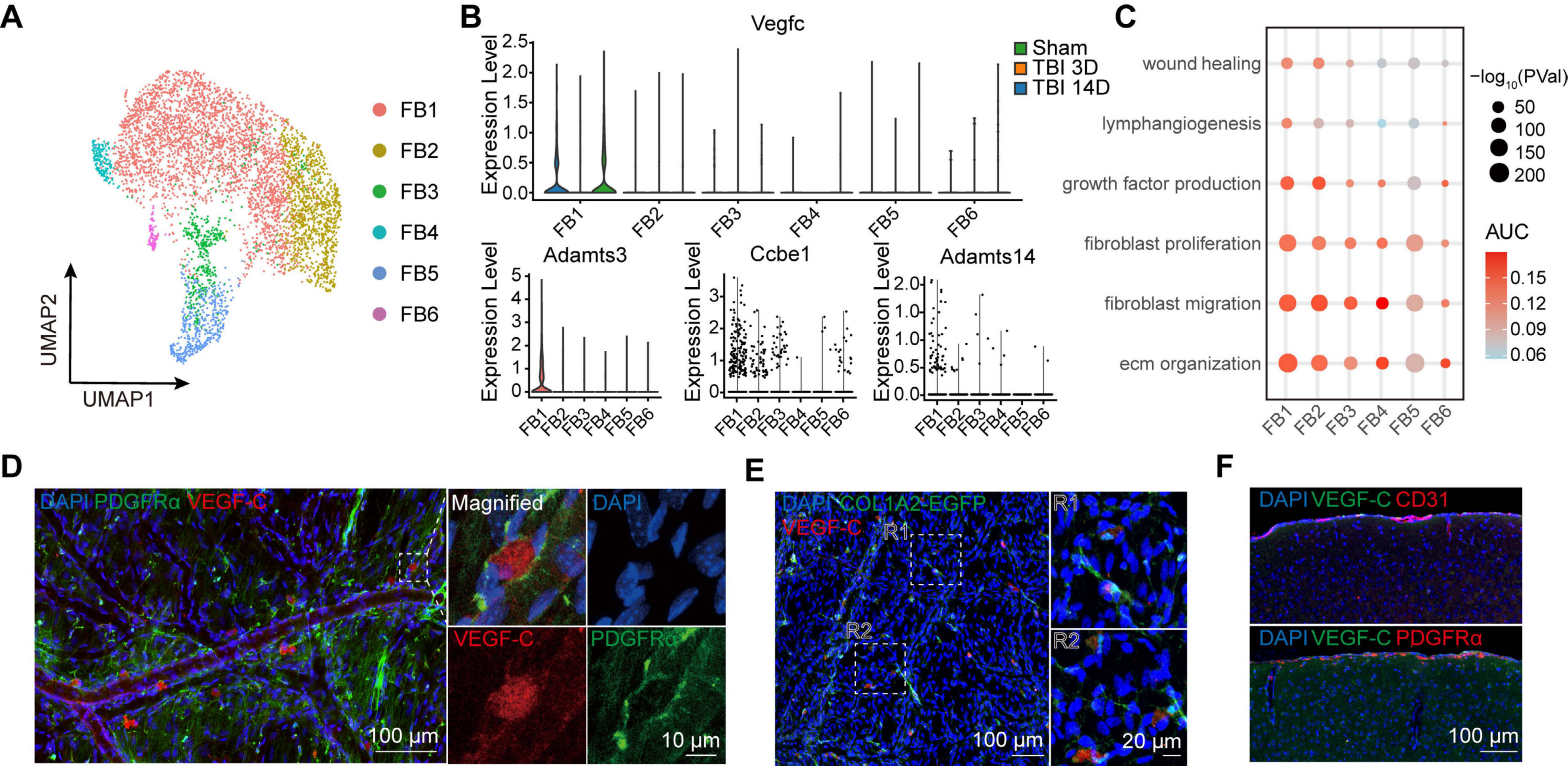
** VEGF**-**C is predominantly synthesized and secreted by meningeal fibroblasts.** (A) UMAP visualization of 6 subsets of fibroblasts. (B) Violin plots showing the expression levels of *Vegfc*, *Adamts3*, *Ccbe1*, and *Adamts14*. (C) Dot plot illustrating functional changes across fibroblast subsets, as inferred from AUCell scores of gene sets provided in Supplementary Data 1. (D) Immunofluorescence showing the co-localization of PDGFRα and VEGF-C. (E) Immunofluorescence showing co-localization of Col1a2-labeled fibroblasts (via AAV-mCol1a2-EGFP) and VEGF-C. (F) Representative immunofluorescence showing VEGF-C⁺ cells within the brain. FB: fibroblast.

To further investigate the characteristics of meningeal fibroblasts, we annotated 6 subgroups for detailed analysis (Figure 2A). The FB1 cluster was identified as the primary source of VEGF-C secretion (Figure S2A), and the transcriptional trend of *Vegfc* was consistent with the qPCR results, exhibiting a decline after TBI (Figure 2B). VEGF-C is initially produced as an inactive pre-pro protein and requires two sequential proteolytic cleavages to be converted into its mature, active form 29. This maturation process is dependent on the metalloproteinase ADAMTS-3 and its cofactor, CCBE1. Furthermore, ADAMTS-14 has been reported to facilitate the proteolytic activation of VEGF-C 11. Accordingly, we assessed the expression patterns of *Adamts3*, *Ccbe1*, and *Adamts14* by scRNA-seq analysis (Figure 2B, Figure S2B). Among the 6 fibroblast subclusters, FB1 exhibited the strongest expression of* Adamts3* and* Ccbe1*, while these genes were detected at very low levels in other meningeal cell populations. By calculating AUCell scores to explore functional changes at the single-cell level, we found that FB1 exhibited stronger capacities in extracellular matrix (ECM) organization, lymphangiogenesis, fibroblast migration, and fibroblast proliferation (Figure 2C). Differential gene expression analysis between FB1 and FB2 was conducted, followed by GO analysis (Figure S2C, D). These findings highlighted the pivotal role of FB1 in ECM remodeling and injury repair. To characterize the VEGF-C⁺ fibroblast population, we stained meninges with an antibody against PDGFRα and labeled fibroblasts using adeno-associated virus 9 (AAV9-mCOL1A2-EGFP). Immunofluorescence staining further confirmed the presence of VEGF-C⁺PDGFRα⁺ and VEGF-C⁺COL1A2⁺ fibroblasts in the meninges (Figure 2D, E). Besides, we examined potential sources of VEGF-C within the brain parenchyma and found that their expression was confined to the leptomeninges (Figure 2F, Figure S2E-F).

### TBI induces extensive changes in meningeal macrophages

**Figure 3:**
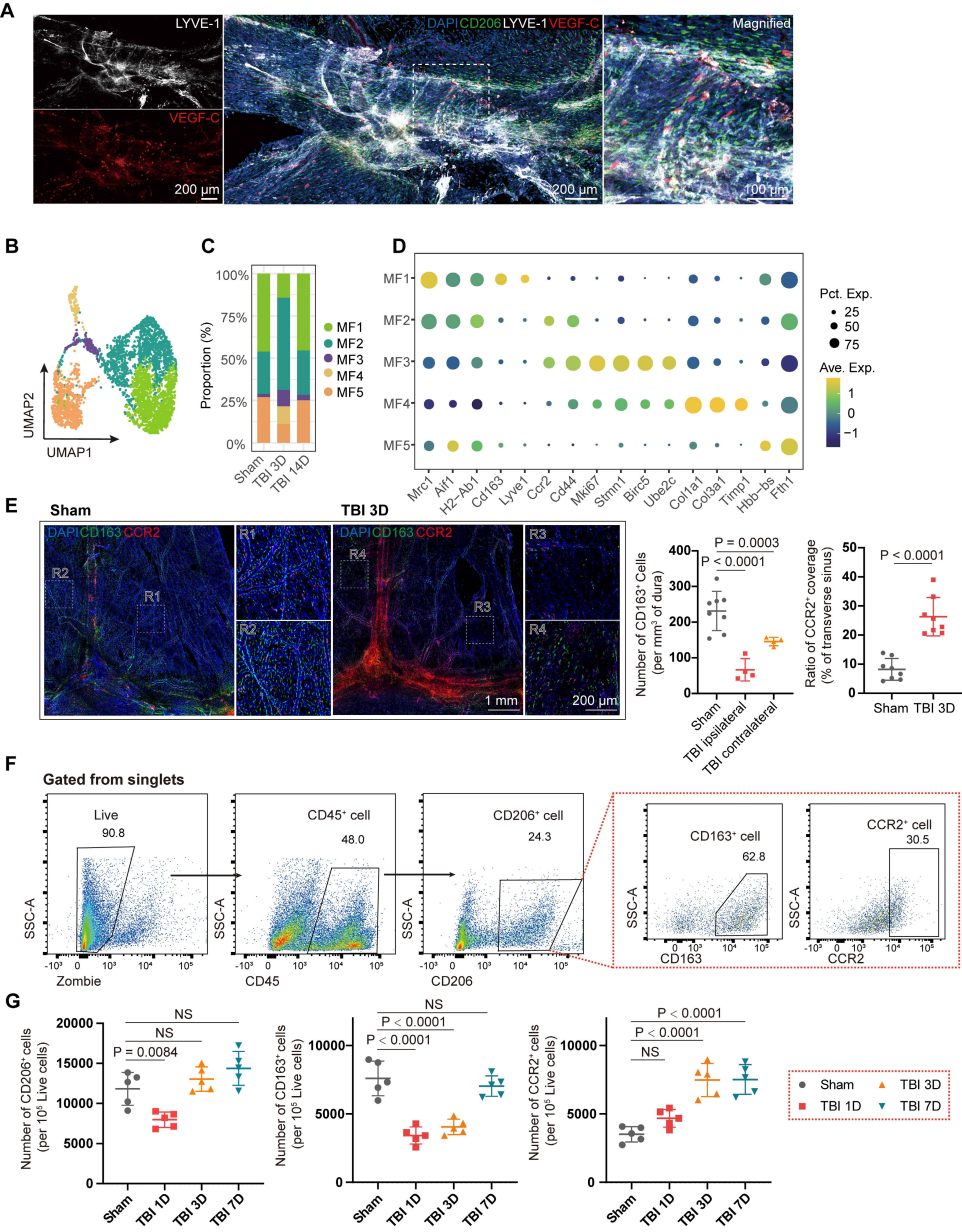
** TBI induces extensive changes of meningeal macrophages.** (A) Representative immunofluorescence images depicting the spatial relationship among mLVs (LYVE-1), macrophages (CD206), and VEGF-C⁺ fibroblasts. (B, C) UMAP visualization and stacked bar plot showing macrophage subsets. (D) Dot plots showing characteristic marker genes in macrophage subsets, with average expression (color) and proportion of expressing cells (dot size). (E) Representative images and quantification of meninges from mice in sham group and those at 3 days post-TBI, staining for CD163 and CCR2 (n = 4, bilateral). (F, G) Gating strategy and quantification of meningeal macrophages by flow cytometry in sham and TBI mice (n = 5). MF: macrophage.

Fibroblasts and macrophages are ubiquitously present across tissues, and accumulating evidence indicates that these two cell types engage in direct and dynamic intercellular communication 20,30,31. By secretion of cytokines and growth factors, including TGF-β1, IL-1β, and PDGF-C, macrophages critically modulate fibroblast activation, fibrotic responses, and tissue repair processes 21,32,33. Macrophages represented the most abundant immune cells in the meninges and underwent the most significant quantitative shifts in the early post-TBI period (Figure 1E). Immunofluorescence in the sinus region showed that VEGF-C⁺ fibroblasts were distributed adjacent to macrophages, suggesting potential interactions between macrophages and fibroblasts (Figure 3A). The heatmap of interaction strengths also indicated significant crosstalk between macrophages and fibroblasts (Figure S3A). Therefore, we isolated meningeal macrophages and performed subclustering analyses to further characterize their transcriptional heterogeneity and dynamic phenotypic changes following TBI.

Macrophages were classified into five subclusters using unsupervised clustering (Figure 3B). At the 3-day time point after TBI, the proportion of meningeal macrophages showed a marked change (Figure 3C). First, we observed a significant decrease in the proportion of MF1 subcluster at 3 days after TBI, which was identified as meningeal-resident macrophages by their high expression of scavenger receptors, such as *Lyve1* and *Cd163* (Figure 3D, Figure S3D). In contrast, a marked increase in the proportion of skull bone marrow-derived macrophages MF2 within the meninges, characterized by high expression of *Ccr2* and *Cd44*, was observed. GO analysis of MF1 and MF2 indicated differences between the two clusters in immune chemotaxis and regulation, and wound healing (Figure S3B-C). Besides, the proportions of MF3 (*Mki67*, *Stmn1*, *Birc5*, and *Ube2c*), MF4 (*Col1a1*, *Timp1*, and *Lox*), and MF5 (*Fth1* and *Hbb-bs*) exhibited significant changes. Notably, MF4 was not detected in the sham group, suggesting that this macrophage population is associated with acute injury. The highly expressed genes in this subset suggest a potential role in ECM remodeling and fibrosis.

Subsequently, we performed immunofluorescence staining with CD163 and CCR2 to label MF1 and MF2 macrophages, respectively, and quantified alterations in the two subclusters (Figure 3E). A marked reduction in CD163⁺ cells was observed at the injured site, accompanied by morphological changes in macrophages on both sides, suggesting widespread alterations in meningeal macrophages following TBI. Besides, CCR2⁺ coverage of the venous sinus region in the TBI group was significantly increased compared with that in the sham group. We further validated these changes in meningeal macrophage subclusters after TBI using flow cytometry at 1, 3, and 7 days after TBI (Figure 3F). In the early stage after TBI, the number of CD45⁺CD206⁺CD163⁺ macrophages markedly decreased, consistent with scRNA-seq and immunofluorescence staining results (Figure 3G). At the 7-day time point, the abundance of CD163⁺ macrophages returned to baseline levels. The pseudotime trajectory inferred by Monocle2 showed that MF1 could be replenished via transitions from MF2 and MF3 (Figure S3D-F), consistent with previous findings 34.

### Meningeal macrophage depletion impairs meningeal lymphatic function

**Figure 4:**
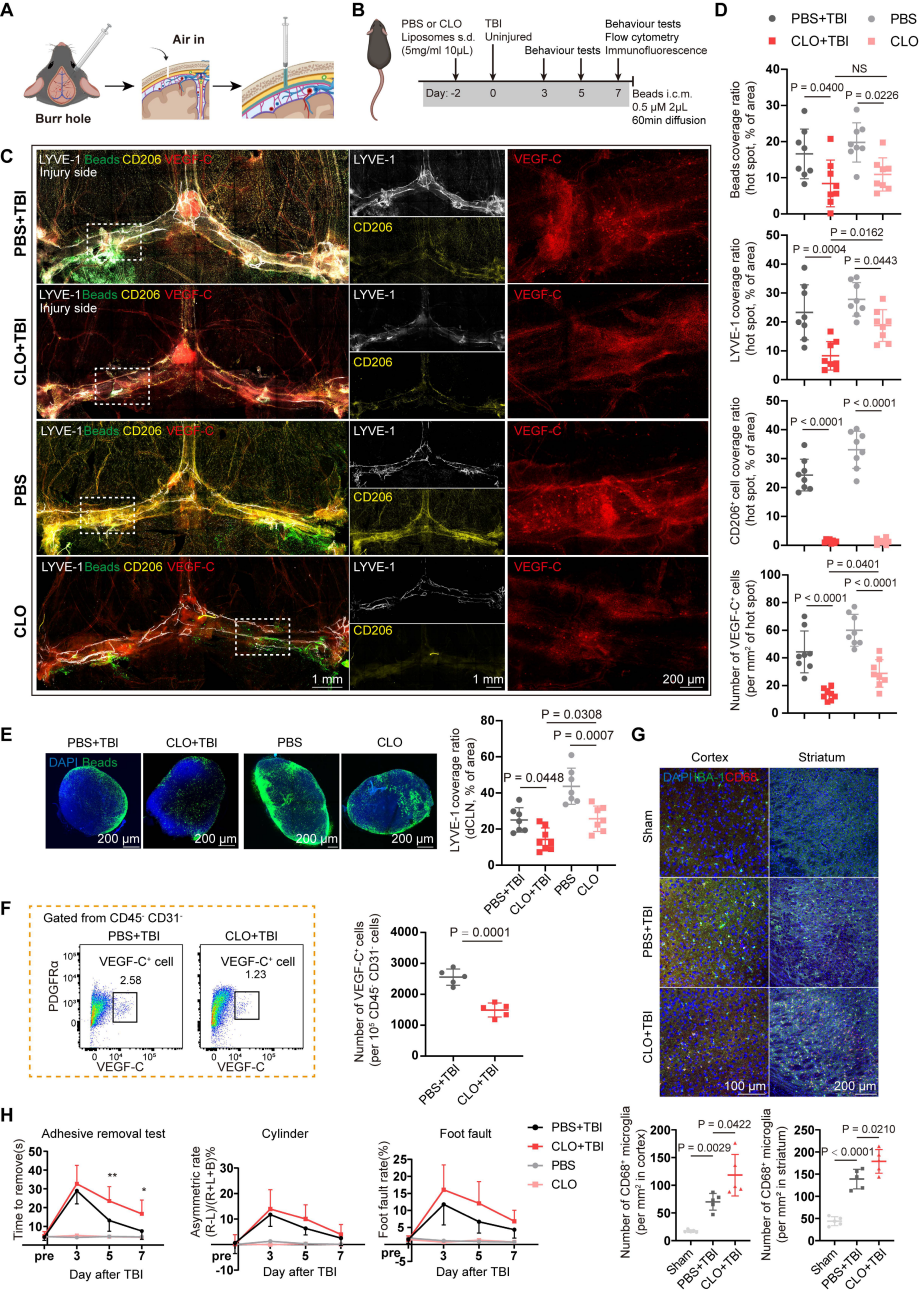
** Depletion of meningeal macrophages impairs meningeal lymphatic function and exacerbates intracerebral inflammation.** (A) Schematic for subdural injection. (B) Workflow for TBI mice with subdural injection of CLO or PBS. (C, D) Immunofluorescence showing mLVs (LYVE-1), beads, macrophages (CD206), and VEGF-C⁺ cells in the meninges of TBI or uninjured mice after subdural injection of CLO or PBS and quantification of bead coverage, LYVE-1 coverage, CD206⁺ macrophages coverage, and VEGF-C⁺ cell numbers in PBS + TBI, CLO + TBI, PBS, and CLO groups (n = 4, bilateral). (E) Representative images and quantification of fluorescent beads accumulation in dCLNs (n = 7 for PBS + TBI, PBS, and CLO group; n= 8 for CLO + TBI group). (F) Representative flow cytometry plots and quantification of CD45⁻CD31⁻VEGF-C⁺ cells from TBI mice following subdural injection of CLO or PBS (n = 5). (G) Representative images and quantification of activated microglia (CD68⁺IBA-1⁺) within cortex and striatum (n = 5). (H) Sensorimotor function assessment in PBS + TBI, CLO + TBI, PBS, and CLO groups at 3, 5, and 7 days post-TBI using the cylinder test, foot fault test, and adhesive removal test (n = 10 for PBS + TBI and CLO + TBI groups; n = 6 for PBS and CLO groups). * PBS + TBI vs. CLO + TBI. * *P* < 0.05, ** *P* < 0.01, *** *P* < 0.001, **** *P* < 0.0001.

Current research on the meninges has been limited by the lack of effective intervention methods. While i.c.m. injection is the predominant method for meningeal targeting, its delivery is largely confined to the perisinusal regions and associated lymphatic vasculature 35. Therefore, we developed a simple yet rarely used subdural injection technique that may provide valuable insights into the meninges and mLVs (Figure 4A). In this study, we performed subdural injections of OVA (45 kDa) and beads (1 μm) within the subdural space to simulate the flow of small molecules and liposomes, respectively. Both OVA and beads readily diffused throughout the entire dura mater within 1 hour after unilateral injection. In contrast, subarachnoid injection led to diffusion restricted to the ipsilateral side (Figure S4A).

To clarify the regulatory effects of macrophages on fibroblasts, we depleted meningeal macrophages using subdural injections of CLO or PBS liposomes serving as controls. Subdural injection of CLO can effectively deplete meningeal macrophages, with minimal effects on border-associated macrophages and microglia in the parenchyma (Figure S4B-F). Besides, depletion of meningeal macrophages markedly reduced the abundance of VEGF-C⁺ cells in the meninges (Figure S4G-H), which was consistent with the scRNA-seq results that macrophages can regulate fibroblasts.

Next, we investigated the effects of meningeal macrophage depletion on the meningeal lymphatic system following TBI. We selected the 7-day post-TBI time point to examine how impaired meningeal lymphatic drainage promotes brain inflammation, as morphological changes in mLVs begin to emerge at this stage. Meningeal macrophages were depleted with CLO two days prior to TBI or in uninjured mice, and beads were administered i.c.m. one hour before tissue harvesting to evaluate meningeal lymphatic function (Figure 4B). Compared with the PBS- and PBS + TBI-treated controls, both CLO- treated mice and CLO + TBI-treated mice exhibited reduced bead aggregation in mLVs and dCLNs (Figure 4C-E). In addition, mice in the CLO + TBI group exhibited features similar to those observed following mLV ablation. Quantitative analysis further revealed a markedly reduced mLV coverage at hot spots compared with the PBS + TBI. Notably, the remaining macrophages clustered around the residual mLVs, which further supports the close relationship between macrophages and mLVs. Then, we quantified meningeal VEGF-C⁺ cells using flow cytometry after CLO + TBI or PBS + TBI treatment. The results showed that macrophage depletion significantly reduced the abundance of meningeal VEGF-C⁺ cells (Figure 4F, Figure S4I-J). Moreover, macrophage depletion led to higher PDGFRα expression in CD45⁻CD31⁻cells (Figure S4K), which may be accounted for by the reduction of PDGF-C caused by macrophage depletion.

### Meningeal macrophage depletion exacerbates intracerebral inflammation

To assess the effects of lymphatic dysfunction induced by macrophages depletion on intracerebral inflammation after TBI, we applied a flow cytometry panel adapted from our previously described protocol to profile multiple immune cell types in the injured hemisphere 7 days post-TBI, including CD45^high^CD11b^-^CD11c^-^ lymphocyte, CD8^+^ T cells, CD4^+^ T cells, CD19^+^ B cells, CD45highCD11b^+^ myeloid cells, LY6G^-^ monocytes/macrophages (MMs), CD11b^+^Ly6G^+^ neutrophils, CD45^high^CD11b^-^CD11c^+^ dendritic cells (DCs), and D45^meduim^CD11b^+^ microglia (Figure S5A) 25.

Meningeal macrophage depletion significantly increased the proportion of infiltrating lymphoid cells (Figure S5B), primarily due to the elevated abundance of CD4⁺ and CD8⁺ T cells (Figure S5C-E). In addition, a delayed increase in neutrophil abundance was observed (Figure S5F). In contrast, no significant changes were detected in brain-infiltrating MMs, microglia, or DCs (Figure S5G-J). These results suggested that macrophage depletion can lead to impaired meningeal lymphatic drainage, which in turn promotes more severe inflammation, particularly antigen-specific inflammation.

Flow cytometric analysis revealed no significant change in the overall number of microglia, the primary resident immune cells of the brain parenchyma. Accordingly, their polarization status was evaluated. Notably, microglial activation, as indicated by CD68⁺IBA-1⁺ cells, was significantly increased in the primary motor cortex and the striatum in the CLO + TBI group (Figure 4G, Figure S5K-L). However, depletion of meningeal macrophages induced by CLO in healthy mice did not directly exacerbate intracerebral inflammation (Figure S5M-Q).

Next, we investigated the association between this condition and behavioral impairments following TBI. Neurological function in mice was assessed during the subacute phase (≤7 days post-TBI) using a battery of tests, including the cylinder test, adhesive removal test, and foot fault test (Figure 4H). Compared with the PBS + TBI group, the CLO + TBI group showed a decrease in performance on the adhesive removal test at 5 and 7 days after TBI, indicating that macrophage depletion impairs sensory-motor functional recovery following TBI, whereas the cylinder test and foot fault test showed some differences but did not reach statistical significance. Moreover, macrophage depletion in healthy mice did not result in significant behavioral changes.

### PDGF-C enhances VEGF-C production and promotes the recovery of meningeal lymphatic function

**Figure 5:**
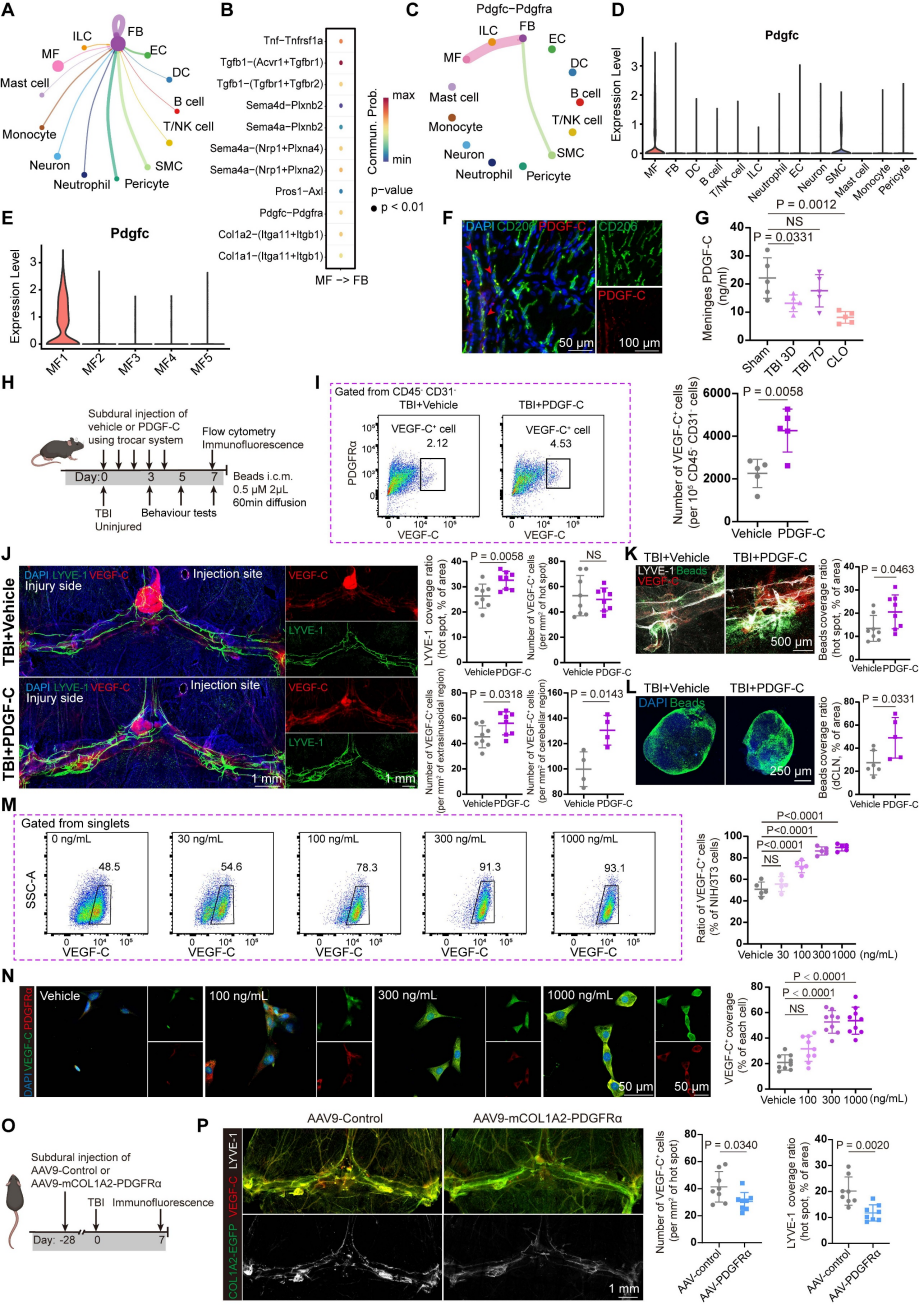
** PDGF-C enhances VEGF-C production by meningeal fibroblasts and promotes the recovery of meningeal lymphatic function after TBI.** (A) Schematic of the signaling network through which other cell types regulate meningeal fibroblasts. (B) Dot plot showing macrophage-fibroblast interactions through ligand-receptor pairs, quantified by ligand-receptor scores. (C) *Pdgfc/Pdgfra* signaling network from other cells to fibroblasts. (D) Violin plots showing the expression levels of *Pdgfc* across cell types. (E) Violin plots showing the expression levels of *Pdgfc* in macrophage subsets. (F) Immunofluorescence staining showed colocalization of PDGF-C with macrophages (CD206). (G) ELISA analysis showing PDGF-C levels after TBI and macrophage depletion (n = 5). (H) Experimental design for TBI and uninjured mice with subdural injection of PDGF-C or vehicle. (I) Representative flow cytometry plots and quantification of CD45⁻CD31⁻VEGF-C⁺ cells from TBI mice following subdural injection of PDGF-C or vehicle (n = 5). (J) Immunofluorescence showing mLVs (LYVE-1) and VEGF-C⁺ cells in the meninges of TBI mice after subdural injection of PDGF-C or vehicle and quantification of LYVE-1 coverage, and VEGF-C⁺ cell abundance in these two groups (n = 4). (K) Representative images and quantification of fluorescent beads accumulation in hot spots (n = 4). (L) Representative images and quantification of fluorescent beads accumulation in dCLNs (n = 6 for TBI + Vehicle, n = 5 for TBI + PDGF-C). (M) Representative flow cytometry plots and quantification of the proportion of VEGF-C⁺ cells in NIH/3T3 fibroblasts treated with different concentrations of PDGF-C *in vitro* (n = 5). (N) Representative images and quantification of VEGF-C⁺ cells in NIH/3T3 fibroblasts treated with different concentrations of PDGF-C *in vitro* (n = 9). (O) Timeline of experimental design illustrating the viral delivery and TBI induction. (P) Representative immunofluorescence images and quantification showing the number of VEGF-C⁺ cells and LYVE-1 coverage in meninges isolated from TBI mice following AAV9 treatment (n = 4).

Given the impact of meningeal macrophage depletion on fibroblasts and the function of mLVs, we further investigated the mechanisms by which macrophages regulate fibroblasts. Incoming communication patterns of fibroblasts indicate that macrophages are important regulators of meningeal fibroblasts (Figure 5A). Cell-cell communication analysis indicated that macrophage-to-fibroblast signaling is predominantly mediated by *Tgfb1*, *Tnf*, *Sema4a*/*Sema4d*, and *Pdgfc* (Figure 5B). Among these pathways, *Tgfb1*, *Tnf*, and *Sema4a*/*Sema4d* are likely involved in fibrotic processes and fibroblast activation 36-38. Previous studies have demonstrated that PDGF-C/PDGFRα is associated with post-injury repair processes and VEGF-C production 11,12,39,40. In light of this, the PDGF-C/PDGFRα signaling pathway was selected for further analysis. Notably, cell-cell communication analysis identified PDGF-C/PDGFRα signaling as a pathway predominantly active in macrophage-fibroblast interactions (Figure 5C). We then assessed PDGF-C expression among meningeal cell populations and macrophage subsets (Figure 5D-E). Notably, *Pdgfc* expression was predominantly localized to MF1 cells and was significantly reduced during acute injury (Figure 3C), consistent with our hypothesis. Based on our sequencing results and support from previous studies, we further validated by immunofluorescence that PDGF-C is predominantly secreted by macrophages (Figure 5F). ELISA analysis also confirmed that PDGF-C levels were significantly reduced following TBI and macrophage depletion (Figure 5G). In addition, PDGF-A, another ligand of PDGFRα, exhibited low-level expression in the meninges, and cell-cell communication analysis indicated that the PDGF-A/PDGFRα interaction was weaker than the PDGF-C/PDGFRα interaction (Figure S6A-B). These findings collectively provided a basis for exploring the potential regulatory effects of meningeal macrophages on fibroblasts.

Next, we investigated whether exogenous PDGF-C administration affects meningeal lymphatic function. For repeated PDGF-C delivery over five consecutive days, a previously characterized trocar system for multiple subdural injections was employed to reduce procedural injury (Figure 5H) 25. We first evaluated the abundance of VEGF-C⁺ cells in the meninges following PDGF-C treatment using flow cytometry. The results showed that PDGF-C intervention approximately doubled the abundance of VEGF-C⁺ cells in the meninges after TBI compared with vehicle treatment (Figure 5I). We next employed immunofluorescence to directly visualize the effects of PDGF-C treatment on VEGF-C production and mLV morphology. The results showed that the TBI + PDGF-C group exhibited greater LYVE-1 coverage and an increased number of lymphatic sprouts (Figure 5J). While the number of VEGF-C⁺ cells in hotspot regions showed no significant difference, a marked increase in VEGF-C⁺ cells was observed at perisinus regions in the TBI + PDGF-C group compared with Vehicle group (Figure 5J). Apart from morphological changes, mice receiving PDGF-C injection exhibited increased bead aggregation in both mLVs and dCLNs (Figure 5K-L). We further evaluated the effects of subdural PDGF-C injection in healthy mice. The results showed no significant differences in LYVE-1 coverage, the number of VEGF-C⁺ cells, or bead aggregation at hot spot regions between Vehicle group and PDGF-C group (Figure S6C, D). In contrast, the abundance of VEGF-C⁺ cells in extrasinusoidal regions was significantly increased. Moreover, bead aggregation in dCLNs did not differ between the two groups (Figure S6E, F).

We subsequently validated the stimulatory effects of PDGF-C on VEGF-C production in fibroblasts *in vitro*. The NIH/3T3 fibroblast cell line was selected for these experiments based on its documented expression of pro-VEGF-C 41. PDGF-C was added to cultures of NIH/3T3 cells, and VEGF-C expression was evaluated by flow cytometry and immunofluorescent staining. We found that PDGF-C treatment resulted in a highly significant concentration-dependent increase in VEGF-C levels (Figure 5M-N). PDGF-C intervention did not result in measurable changes in NIH/3T3 cell viability, as determined by the CCK-8 assay (Figure S6G).

Finally, we employed an AAV9 vector carrying the *Col1a2* promoter, which enabled specific targeting of meningeal fibroblasts to induce PDGFRα knockdown (Figure 5O) 42,43. The EGFP fluorescence signal from AAV9 indicated that the virus can specifically target meningeal fibroblasts. In this study, mice were randomly divided into two groups and received either the control virus (AAV9-control) or the PDGFRα-silencing virus (AAV9-mCOL1A2-PDGFRα). TBI was induced after 28 days of viral expression. Immunofluorescence analysis indicated that PDGFRα was effectively knocked down in fibroblasts (Figure S6H), which was accompanied by a reduction in the abundance of VEGF-C⁺ cells and a decreased coverage area of mLVs at 7 days post-TBI (Figure 5P).

### PDGF-C-mediated recovery of meningeal lymphatic function improves post-TBI neuroinflammation and neurological outcomes

**Figure 6:**
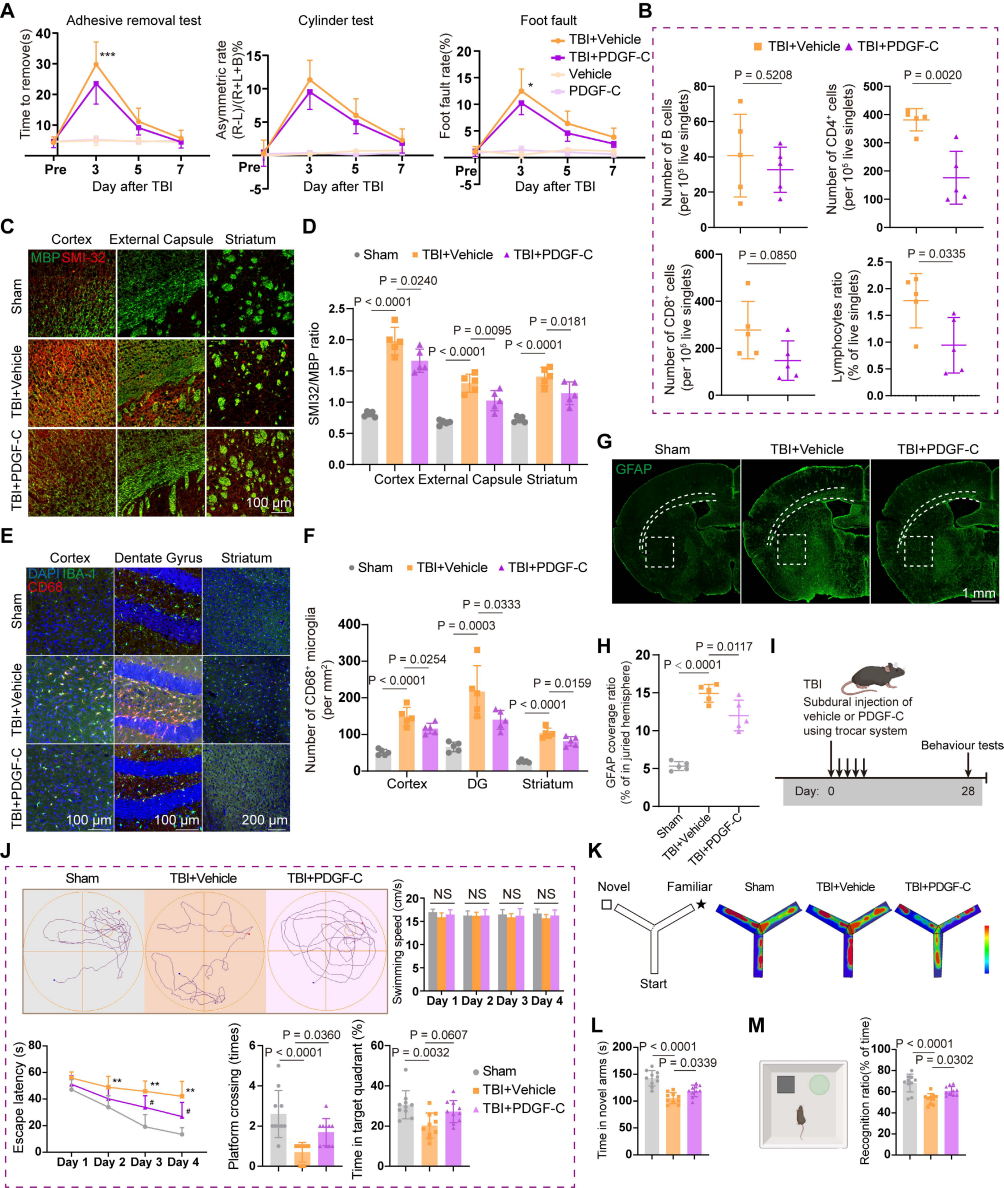
** PDGF**-**C**-**mediated recovery of meningeal lymphatic function improves post**-**TBI neuroinflammation and neurological outcomes**. (A) Sensorimotor function assessment in TBI + Vehicle, TBI + PDGF-C, Vehicle, and PDGF-C groups at 3, 5, and 7 days post-TBI using the adhesive removal test, cylinder test, and foot fault test (n = 10 for TBI + Vehicle and TBI + PDGF-C groups; n = 6 for Vehicle and PDGF-C groups). (B) Changes in the proportion and number of infiltrating lymphoid cells within the injured hemisphere (n = 5). (C, D) Representative images and quantification of MBP and SMI32 coverage in the injured hemisphere at 7 days post-TBI (n = 5). (E, F) Representative images and quantification of activated microglia (CD68⁺IBA-1⁺) in the cortex, dentate gyrus of hippocampus and striatum (n = 5). (G, H) Representative images and quantification of GFAP (green) coverage in the injured hemisphere (n = 5). (I) Experimental design for long-term memory and cognitive assessment in TBI + PDGF-C and TBI + Vehicle groups at 28 days post-TBI (n = 10) (J) Representative path tracings during the probe trial, and quantification of swimming speed, escape latency, platform crossings, and time spent in target quadrant in Morris water maze test (n = 10). * TBI + vehicle vs. sham; # TBI + PDGF-C vs. TBI + vehicle. (K, L) Schematic and representative heatmaps during testing, and quantification of time spent in the novel arms in Y-maze test (n = 10). (M) Schematic and quantification of the recognition ratio in the novel object recognition test (n = 10). * *P* < 0.05, ** *P* < 0.01, # *P* < 0.05.

We next sought to explore the effects of PDGF-C-mediated recovery of meningeal lymphatic function on neuroinflammation and neurological outcomes. Accordingly, sensorimotor function was assessed in mice within 7 days after TBI and in healthy mice (Figure 6A). The results indicated that PDGF-C treatment significantly improved performance in the foot fault and adhesive removal test at 3 days post-TBI, although no overall differences were observed in the cylinder test (Figure 6A). Overall, these findings revealed that PDGF-C treatment promoted the recovery of sensorimotor function after TBI. Moreover, flow cytometry analysis of brain hemispheres at 7 days post-TBI revealed reduced lymphocyte infiltration in the PDGF-C group, primarily due to decreased CD4⁺ T cells (Figure 6B). Besides, other immune cell populations did not show significant alterations (Figure 6B, Figure S7A-E).

Recent evidence indicates that meningeal lymphatic function is associated with myelin repair and regeneration 44. Considering that TBI not only causes damage to the grey matter but also induces injury to multiple white matter nuclei and axons, we further evaluated the effects of PDGF-C treatment on white matter and myelin after TBI. The results showed that the SMI32/MBP ratio in the cortex, dentate gyrus, and striatum was significantly reduced in the PDGF-C group, suggesting that promoting meningeal lymphatic function may facilitate myelin regeneration after TBI (Figure 6C-D).

We subsequently evaluated CD68⁺IBA-1⁺ markers to assess microglial activation in the cortex, dentate gyrus, striatum, and cingulum (Figure 6E, Figure S7F). The results indicated attenuation of widespread brain inflammation (Figure 6F, Figure S7G-H), attributed to PDGF-C-mediated enhancement of mLV function. Besides, at 7 days post-TBI, the GFAP-positive area ratio was significantly higher in the Vehicle group than in the PDGF-C group, indicating robust astrogliosis (Figure 6G-H). In healthy mice, subdural PDGF-C injection did not alter cortical microglial abundance, the proportion of CD68⁺ microglia, or astrocytic coverage (Figure S7I-J). Collectively, these results demonstrated that PDGF-C-mediated recovery of meningeal lymphatic function plays a crucial role in improving neurological outcomes and suppressing intracerebral inflammation.

Finally, we examined the long-term effects of PDGF-C treatment on neurological function at 28 days post-TBI (Figure 6I). The administration was performed using the previously described trocar injection system, with the modification that the system was removed five days after injection. Compared with the Vehicle group, PDGF-C-treated mice exhibited marked recovery of long-term spatial learning and memory in the Morris water maze test (Figure 6J). We further validated the improvement in post-traumatic spatial cognition using the Y-maze test, in which PDGF-C-treated mice spent more time in the novel arm (Figure 6K-L). In the novel object recognition test, PDGF-C-treated mice spent significantly more time exploring the novel object, indicating preserved recognition memory (Figure 6M).

## Discussion

Although the role of VEGF-C in acute brain injury has been extensively studied, little is known about the precise cellular sources of VEGF-C and related regulatory mechanisms, and whether these processes can be regulated 6,45-47. Therefore, further investigation into the origin and regulatory plasticity of VEGF-C is crucial for advancing our comprehensive understanding of tissue repair mechanisms after acute injury. Herein, we identified meningeal fibroblasts as the primary source of VEGF-C and revealed their critical crosstalk with meningeal macrophages in regulating meningeal lymphatic function. Macrophage depletion impaired fibroblast-derived VEGF-C production, resulting in severe deficits in meningeal lymphatic function. We further showed that PDGF-C is a key regulator of meningeal fibroblasts, and subdural injection of PDGF-C after TBI improved lymphatic drainage, white matter integrity, neuroinflammation and cognitive deficits. Moreover, fibroblast-specific PDGFRα knockdown reduced the abundance of VEGF-C⁺ cells and impaired lymphatic function. Overall, these findings offer a novel and promising therapeutic intervention for promoting neurological repair after TBI.

The meninges are a membranous structure that envelops the CNS and provides continuous immune surveillance at the CNS border, where macrophages are the predominant immune cell population 48-50. Meningeal macrophages are a heterogeneous population of immune cells that are often described as dynamic first responders to injury 50. Previous studies have reported a transient reduction of meningeal macrophages at the injury site after TBI, followed by replenishment with peripheral monocytes 51.

However, recent evidence suggested that this replenishment primarily originated from the calvarial immune cell reservoir 34,52. Our scRNA-seq and flow cytometry analyses similarly revealed a rapid decline of meningeal resident macrophages during the acute and subacute phases of TBI, which were replenished by CCR2⁺ macrophages. To the best of our knowledge, tissue-resident macrophages play important roles in angiogenesis, tissue repair, fibrosis regulation, and homeostatic restoration, and their depletion directly reduces the secretion of repair-associated cytokines and exacerbates tissue damage 53-57. Moreover, previous studies have demonstrated that PDGF-C is an important ligand that enables resident macrophages to regulate fibroblasts during tissue repair 33,58,59. Our work consistently showed that meningeal resident macrophages represent a major source of PDGF-C, and that their reduction after TBI can disrupt immune homeostasis, alter fibroblast function, and attenuate lymphatic vessel repair.

PDGF-C, an important member of the PDGF family, serves critical functions in diverse physiological and pathological processes. Recent research has established its essential role in angiogenesis (including promoting endothelial cell proliferation, migration, and vascular stabilization to support neovascularization), primarily through activation of PDGFR-α 60,61. In addition, PDGF-C has been shown to regulate lymphangiogenesis through putative synergistic interactions with the VEGF-C/VEGFR-3 signaling pathway, thereby facilitating lymphatic development 62. Meanwhile, PDGFRα⁺ fibroblasts/mesenchymal cells are increasingly recognized for their contribution to promoting lymphatic development and maintenance by secreting VEGF-C 39,40. Interestingly, a recent study in zebrafish similarly identified VEGF-C production by PDGFRα⁺ fibroblasts at the brain surface 12. Our results consistently demonstrated that PDGFRα⁺ fibroblasts generate VEGF-C under the regulation of meningeal macrophages, a conclusion strongly supported by this finding. The VEGF-C⁺ cells identified in our study may provide valuable insights for future investigations of meningeal lymphatic function. On the one hand, VEGF-C is a central regulator of meningeal lymphangiogenesis and functional maintenance, and identifying its cellular origins is critical for deciphering the molecular mechanisms underlying lymphatic-immune crosstalk in the meningeal microenvironment. On the other hand, meningeal lymphatic dysfunction and immune imbalance are important pathogenic mechanisms in neurological disorders, such as TBI, stroke, and Alzheimer's disease. Tracking VEGF-C-secreting cells may thus provide new insights into the pathogenesis and progression of these diseases.

Enhancing meningeal lymphatic drainage function has been shown to alleviate neuroinflammation by facilitating the clearance of inflammatory mediators 63,64. Moreover, recent studies have revealed that lymphatic dysfunction induces microglia-mediated upregulation of interleukin-6 expression, which subsequently promotes an inhibitory synaptic phenotype and ultimately leads to deficits in memory and cognitive function 65. Consistent with these results, we showed that improved mLV function can alleviate microglial and astrocyte overactivation and reduce T cell infiltration in the injured brain. Another recent work demonstrated that mLVs dysfunction elicits detrimental neuroimmune responses, resulting in oligodendrocyte loss, downregulation of MBP, and demyelination 44. A longitudinal cohort study revealed that impaired glymphatic function in humans is associated with the progression of white matter hyperintensities, whereas in mouse models, enhanced meningeal lymphangiogenesis can attenuate microglial proliferation and white matter demyelination 66. In line with these findings, our results showed that restoration the drainage function of mLVs after TBI preserved myelin integrity and reduced demyelination, highlighting mLVs as a pivotal regulator of both inflammatory resolution and white matter protection. Importantly, these effects culminated in significantly enhanced memory and cognitive function at 30 days post-TBI.

There are several limitations to our study that should be acknowledged. First, several technical challenges were encountered during the establishment of PDGF-C-specific knockout transgenic animal models, which limited our ability to directly validate the unique regulatory role of meningeal resident macrophages in neuroinflammation and lymphatic function. Although our study has provided substantial evidence that meningeal macrophages can regulate fibroblasts through PDGF-C, further research is warranted to comprehensively characterize the alterations of meningeal fibroblasts and mLVs in *Pdgfc*-Cre^ERT2^; Lyve1*^flox/flox^* mice. Second, CLO was employed to deplete meningeal macrophages comprehensively in our study; however, beyond resident meningeal macrophages, other immune subsets may modulate meningeal lymphatic function through alternative mechanisms. Future investigations into the contribution of meningeal fibroblast-derived VEGF-C to the pathogenesis of various acute and chronic neurological disorders will also be essential.

In conclusion, our study identified a specific subset of meningeal fibroblasts as the major source of VEGF-C and demonstrated that this process is regulated by meningeal macrophages. In addition, PDGF-C emerges as a key regulator of meningeal fibroblasts, governing lymphatic drainage by modulating VEGF-C production. Overall, our findings uncover novel mechanisms by which border-associated immunity regulates mLVs after brain injury and highlight this axis as a promising therapeutic target for restoring neuroimmune homeostasis.

## Supplementary Material

Supplementary figures and tables.

## Data Availability

The datasets generated and analyzed during the current study are available from the corresponding author on reasonable request.

## Abbreviations

CNS: central nervous system
mLV: meningeal lymphatic vessel
TBI: traumatic brain injury
CSF: cerebrospinal fluid
LEC: lymphatic endothelial cell
scRNA-seq: single-cell RNA sequencing
CLO: clodronate liposomes
i.c.m.: Intra-cisterna magna
dCLN: deep cervical lymph node
ROI: region of interest
GO: Gene Ontology
qPCR: quantitative polymerase chain reaction
ELISA: enzyme-linked immunosorbent assay
ECM: extracellular matrix
OVA: ovalbumin
MM: monocytes/macrophage
DC: dendritic cell
