# Supplementary material for: Meningeal macrophages regulate fibroblasts to influence meningeal lymphatic function following traumatic brain injury
Source: Theranostics. 2026 Mar 4;16(10):5185–203. doi: 10.7150/thno.130263 (PMC13080450; doi:10.7150/thno.130263)
Supplement: Supplementary file 1 — Supplementary figures and tables. [file thnov16p5185s1.pdf]

Supplementary Materials for

• Meningeal macrophages regulate fibroblasts to influence meningeal lymphatic function following TBI

Xiaoming Guo *et al.*

*Corresponding author. Yuan Hong Email: hy0904@zju.edu

This PDF file includes:

Figs. S1 to S7
Tables S1 to S2

Figure S1 VEGF-C⁺ cells are present in the meninges. Related to Figure 1. (A) Immunofluorescence showing the overall view of the meninges, with VEGF-C^{+} cells located around arteries (Lectin) and veins (EphB4). (B) Representative images of cerebellar meninges isolated from sham and 3 days post-TBI groups, staining for VEGF-C and CD31. (C) Bar plots of sc-RNA analyses showing *Vegfc* expression in EC and FB. EC endothelial cells, FB fibroblast. (D) Representative pseudocolor flow cytometry plots of CD45^{-}CD31^{-}VEGF-C^{+} meningeal cells from sham and 1, 3, 7 days post-TBI mice. FB: fibroblast, EC: endothelial cell.

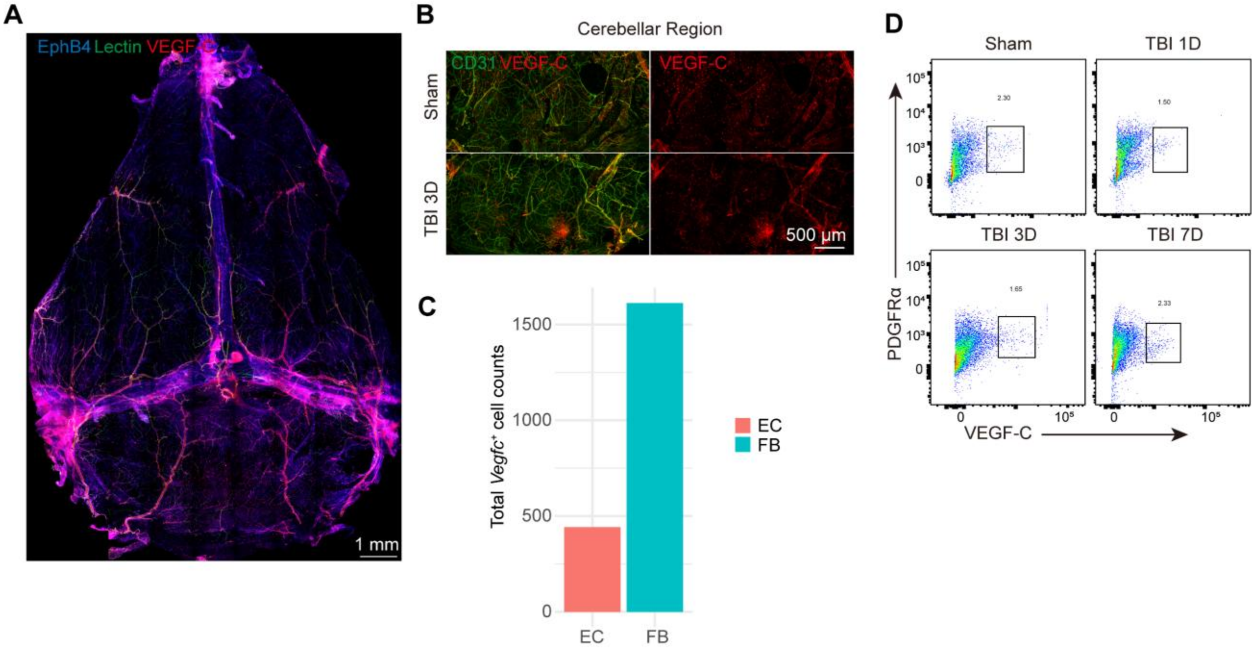

Figure S2 FB1 is the primary cell population responsible for VEGF-C production in the meninges. Related to Figure 2. (A) Violin plots show *Vegfc* expression in FB subsets and EC. FB, fibroblast, EC, endothelial cell. (B) Violin plots showing the expression of *Adamts3*, *Ccbe1*, *Adamts14*, and *Adamts2* in different cell types of the meninges. (C) Volcano plot showing differentially expressed genes between FB1 and FB2. (D) Dot plot showing functional differences in GO enrichment analysis between FB1 and FB2. (E, F) Representative immunofluorescence images showing the absence of VEGF-C^+ cells in the choroid plexus, while a small number of VEGF-C^+ cells are present in leptomeninges. FB: fibroblast.

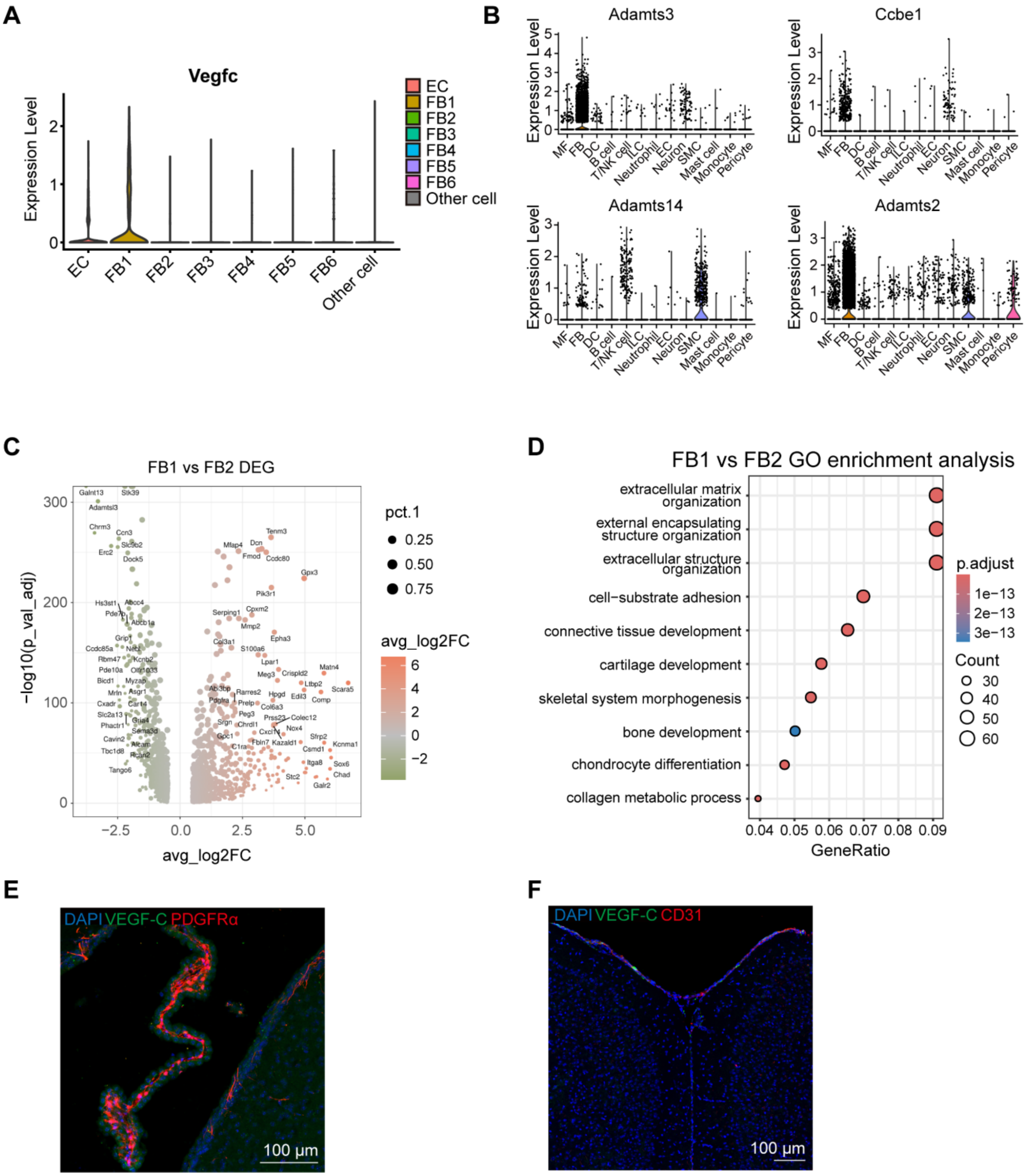
A
Vegfc
Expression Level
EC
FB1
FB2
FB3
FB4
FB5
FB6
Other cell
B
Adamts3
Ccbe1
Adamts14
Adamts2
Expression Level
MF FB DC B cell T/NK cell ILC Neutrophil EC Neuron SMC Mast cell Monocyte Pericyte
C
FB1 vs FB2 DEG
−log10(p_val_adj)
avg_log2FC
pct.1
0.25
0.50
0.75
avg_log2FC
D
FB1 vs FB2 GO enrichment analysis
extracellular matrix organization
external encapsulating structure organization
extracellular structure organization
cell−substrate adhesion
connective tissue development
cartilage development
skeletal system morphogenesis
bone development
chondrocyte differentiation
collagen metabolic process
p.adjust
1e−13
2e−13
3e−13
Count
30
40
50
60
GeneRatio
E
DAPI VEGF-C PDGFRα
100 μm
F
DAPI VEGF-C CD31
100 μm

Figure S3 Multiple macrophage subsets are present in the meninges, which undergo changes following TBI. Related to Figure 3. (A) CellChat analysis showing cell communication between macrophages and fibroblasts. (B) Heatmap showing the top 5 genes of macrophage subsets (MF1-5). (C) Dot plot showing functional differences in GO enrichment analysis between MF1 and MF2. (D, E) Pseudotime trajectory of macrophage subclusters. (F) Gene expression changes in the pseudotime analysis.

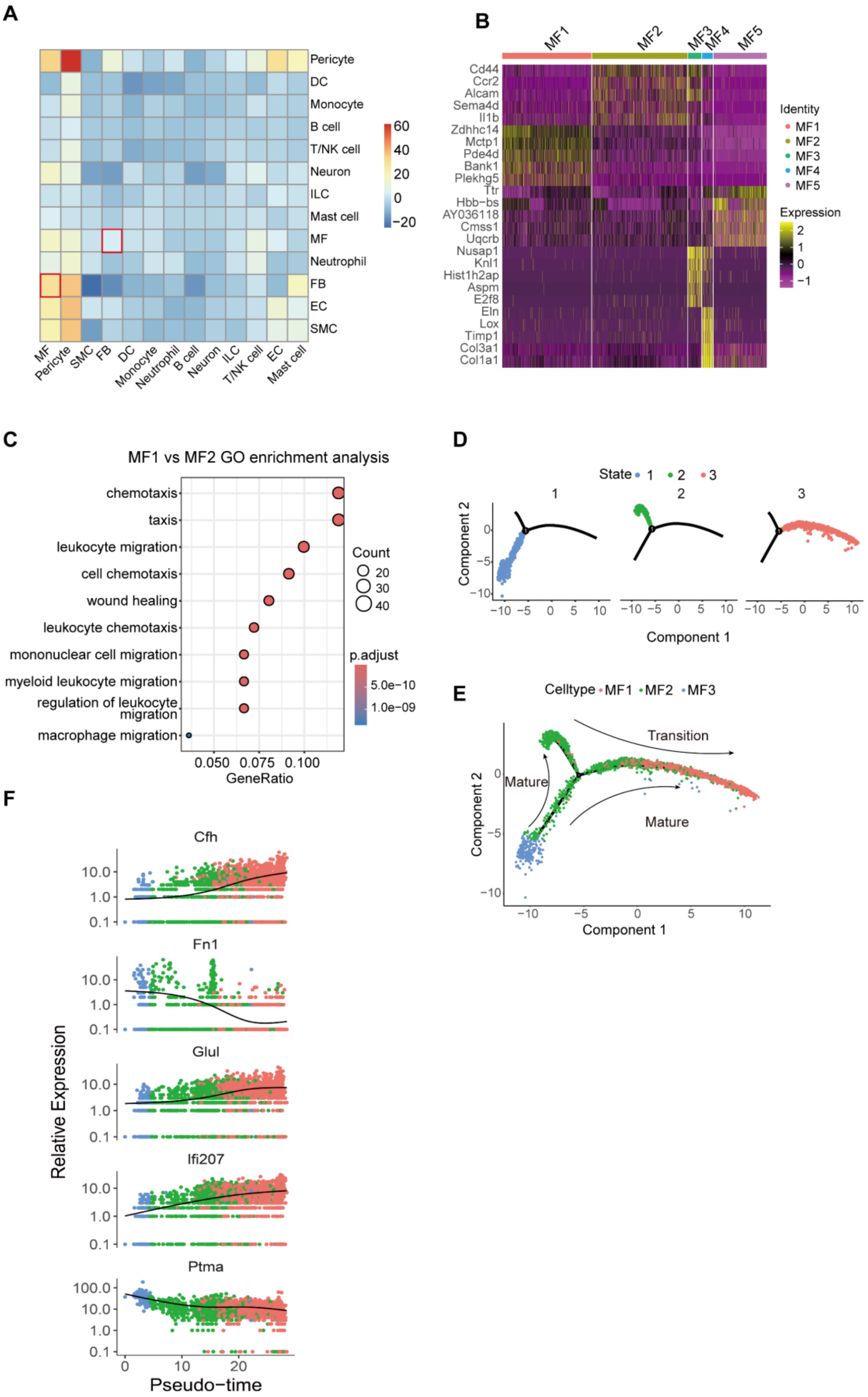
A
Pericyte
DC
Monocyte
B cell
T/NK cell
Neuron
ILC
Mast cell
MF
Neutrophil
FB
EC
SMC
60
40
20
0
−20
B
MF1
MF2
MF3
MF4
MF5
Cd44
Ccr2
Alcam
Sema4d
Il1b
Zdhhc14
Mctp1
Pde4d
Bank1
Plekhg5
Ttr
Hbb−bs
AY036118
Cmss1
Uqcrb
Nusap1
Knl1
Hist1h2ap
Aspm
E2f8
Eln
Lox
Timp1
Col3a1
Col1a1
Identity
Expression
C
MF1 vs MF2 GO enrichment analysis
chemotaxis
taxis
leukocyte migration
cell chemotaxis
wound healing
leukocyte chemotaxis
mononuclear cell migration
myeloid leukocyte migration
regulation of leukocyte migration
macrophage migration
Count
p.adjust
5.0e−10
1.0e−09
GeneRatio
D
State
Component 2
Component 1
E
Celltype
Transition
Mature
Mature
F
Cfh
Fn1
Glul
Ifi207
Ptma
Relative Expression
Pseudo−time

Figure S4 Subdural injection serves as an effective technique for targeted intervention in the dura mater, while exhibiting minimal impact on brain tissue. Related to Figure 4. (A) Representative images showing subdural injection of OVA-647 and fluorescent beads, and subarachnoid injection of OVA-647. (B) Representative images showing depletion of meningeal macrophages following subdural injection of CLO, with only a few residual macrophages remaining (dashed lines in the images). (C-E) Overview of fluorescent images and quantitative analysis of parenchymal macrophages (CD206, green) and microglia (IBA-1, red) changes after PBS and CLO treatment in the brain. (F) Immunofluorescence images showing no changes of macrophages in the choroid plexus (CD206, green) after PBS and CLO treatment. (G, H) Representative images and quantification of VEGF-C^{+} cell changes after PBS and CLO treatment. (I, J) Representative flow cytometry pseudocolor plots and quantification of meningeal macrophages for CLO+TBI and PBS+TBI groups. (K) Flow cytometry histogram and quantitative analysis of changes in PDGFRα expression for CLO+TBI and PBS+TBI groups.

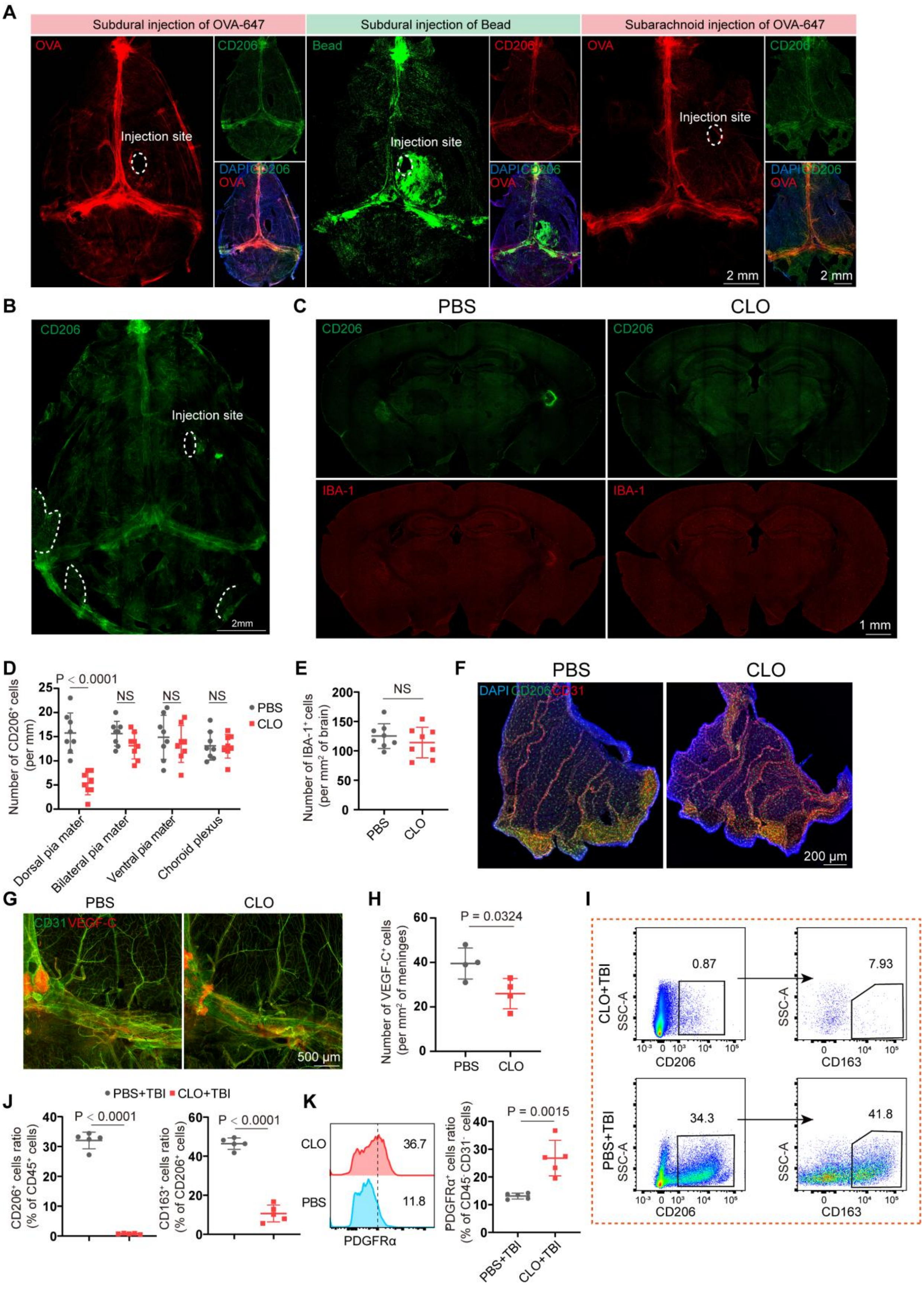
A
Subdural injection of OVA-647
Subdural injection of Bead
Subarachnoid injection of OVA-647
OVA
CD206
Bead
DAPI CD206
Injection site
2 mm
B
C
PBS
CLO
IBA-1
1 mm
2mm
D
P < 0.0001
NS
Number of CD206+ cells (per mm)
Dorsal pia mater
Bilateral pia mater
Ventral pia mater
Choroid plexus
E
Number of IBA-1+ cells (per mm2 of brain)
F
DAPI CD206 CD31
200 μm
G
CD31 VEGF-C
500 μm
H
P = 0.0324
Number of VEGF-C+ cells (per mm2 of meninges)
I
CLO+TBI
PBS+TBI
SSC-A
CD206
CD163
0.87
7.93
34.3
41.8
J
PBS+TBI
CLO+TBI
CD206+ cells ratio (% of CD45+ cells)
CD163+ cells ratio (% of CD206+ cells)
K
36.7
11.8
PDGFRα
P = 0.0015
PDGFRα+ cells ratio (% of CD45-CD31- cells)

Figure S5 Effects of meningeal macrophage depletion on intracerebral inflammation. Related to Figure 4. (A) Flow cytometry gating strategy for immune cells in the brain. (B-J) Quantification of immune cells in PBS+TBI and CLO+TBI groups. (K, L) Quantification of microglia in the cortex and striatum in the sham, PBS+TBI, and CLO+TBI groups. (M-O) Immunofluorescence images and quantification showing that subdural PBS or CLO injection does not alter cortical microglia or $CD68^+$ microglia in healthy mice (n = 4). (P, Q) Immunofluorescence images and quantification showing that subdural PBS or CLO injection does not alter GFAP coverage (n = 4).

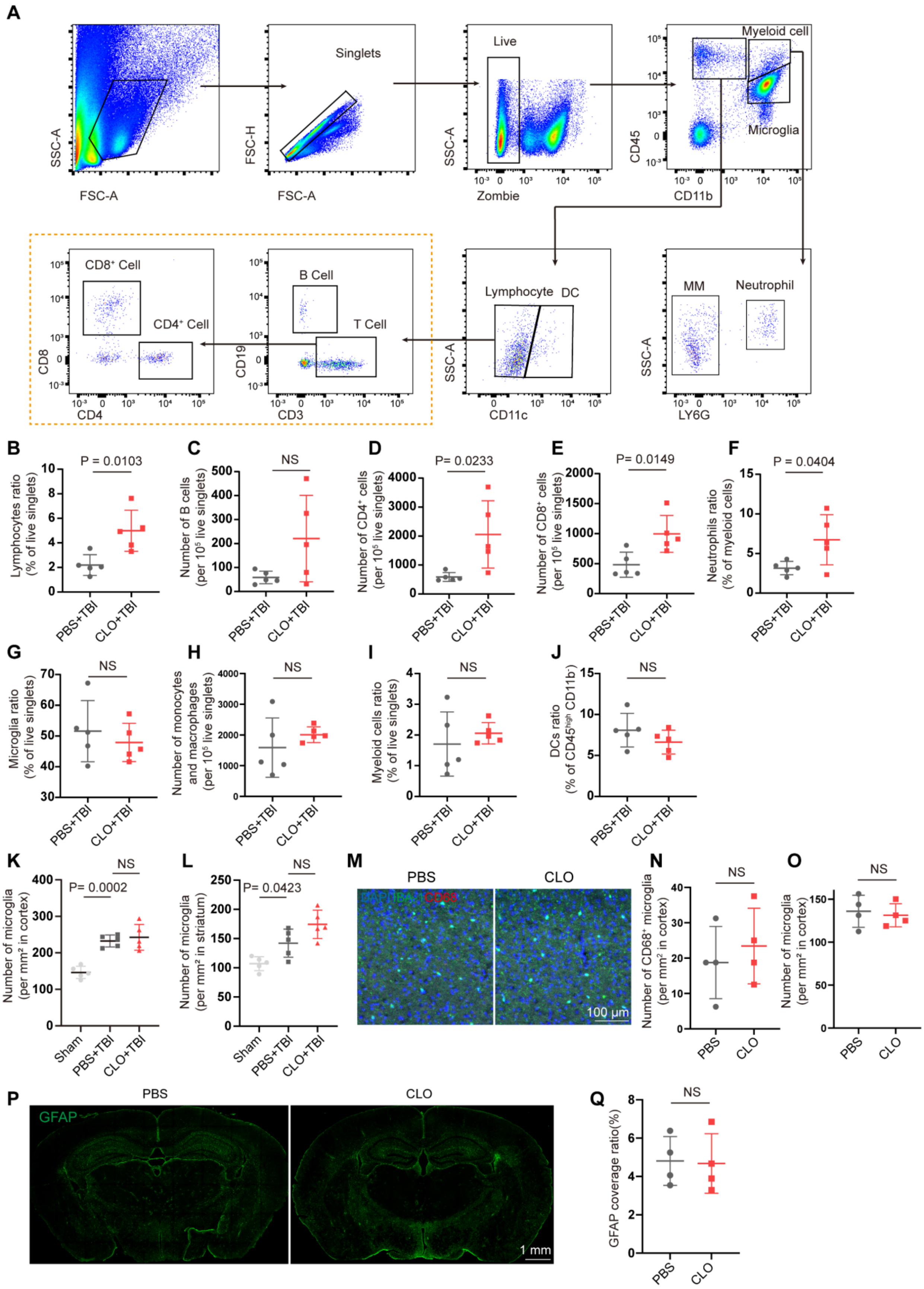
A
Singlets
Live
Myeloid cell
Microglia
SSC-A
FSC-A
FSC-H
Zombie
CD45
CD11b
CD8⁺ Cell
CD4⁺ Cell
B Cell
T Cell
Lymphocyte
DC
MM
Neutrophil
CD8
CD4
CD19
CD3
CD11c
LY6G
B
P = 0.0103
Lymphocytes ratio (% of live singlets)
C
NS
Number of B cells (per 10⁵ live singlets)
D
P= 0.0233
Number of CD4⁺ cells (per 10⁵ live singlets)
E
P= 0.0149
Number of CD8⁺ cells (per 10⁵ live singlets)
F
P = 0.0404
Neutrophils ratio (% of myeloid cells)
PBS+TBI
CLO+TBI
G
NS
Microglia ratio (% of live singlets)
H
NS
Number of monocytes and macrophages (per 10⁵ live singlets)
I
NS
Myeloid cells ratio (% of live singlets)
J
NS
DCs ratio (% of CD45ʰⁱᵍʰ CD11b⁻)
K
P= 0.0002
NS
Number of microglia (per mm² in cortex)
Sham
L
P= 0.0423
NS
Number of microglia (per mm² in striatum)
M
PBS
CLO
100 μm
N
NS
Number of CD68⁺ microglia (per mm² in cortex)
O
NS
Number of microglia (per mm² in cortex)
P
GFAP
1 mm
Q
NS
GFAP coverage ratio(%)

Figure S6 Subdural PDGF-C injection does not alter meningeal lymphatic function in healthy mice. Related to Figure 5. (A) Violin plots show *Pdgfa* expression across cell clusters in the meninges. (B) *Pdgfa/Pdgfra* signaling network from other cells to fibroblasts. (C, D) Immunofluorescence showing mLVs (LYVE-1), Beads, macrophages (CD206), and VEGF-C$^+$ cells in the meninges of uninjured mice after subdural injection of Vehicle or PDGF-C and quantification of bead coverage, LYVE-1 coverage, CD206$^+$ macrophages, and VEGF-C$^+$ cell numbers in Vehicle and PDGF-C groups (n = 4). (E, F) Representative images and quantification of fluorescent beads accumulation in dCLNs (n = 7 for Vehicle group, and n= 6 for PDGF-C group).

(G) Histogram showing cell viability at different concentrations of PDGF-C. (H) Representative immunofluorescence showing changes in PDGFRα and VEGF-C expression after subdural injection of AAV9-Control and AAV9-mCOL1A2-PDGFRα.

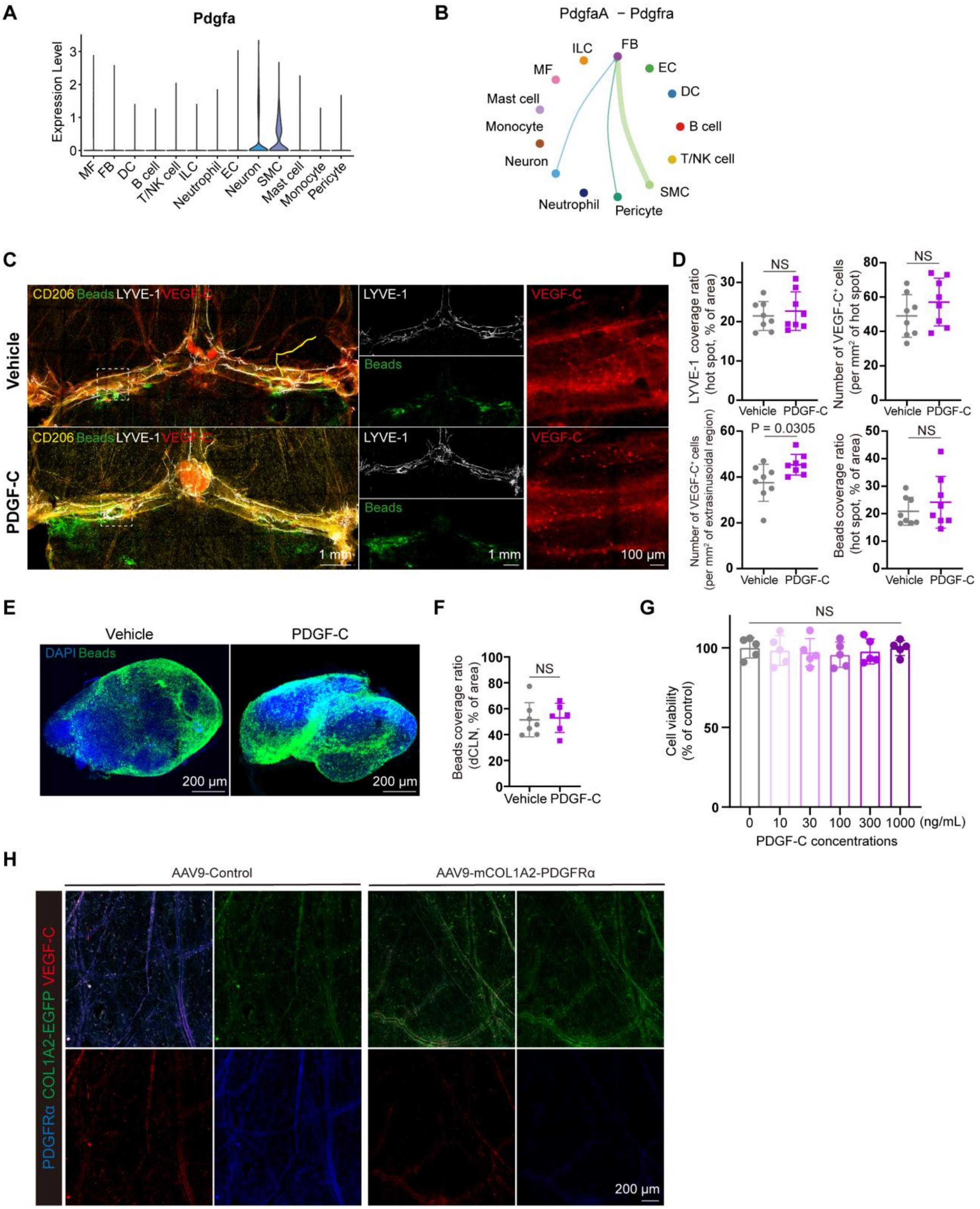

A
Pdgfa
Expression Level
MF FB DC B cell T/NK cell ILC Neutrophil EC Neuron SMC Mast cell Monocyte Pericyte
B
PdgfaA − Pdgfra
FB
ILC
MF
EC
DC
Mast cell
Monocyte
B cell
Neuron
T/NK cell
SMC
Neutrophil
Pericyte
C
CD206 Beads LYVE-1 VEGF-C
LYVE-1
VEGF-C
Vehicle
Beads
PDGF-C
1 mm
100 μm
D
NS
LYVE-1 coverage ratio (hot spot, % of area)
Number of VEGF-C⁺ cells (per mm² of hot spot)
Vehicle PDGF-C
P = 0.0305
Number of VEGF-C⁺ cells (per mm² of extrasinusoidal region)
Beads coverage ratio (hot spot, % of area)
E
Vehicle
PDGF-C
DAPI Beads
200 μm
F
Beads coverage ratio (dCLN, % of area)
G
Cell viability (% of control)
0 10 30 100 300 1000 (ng/mL)
PDGF-C concentrations
H
AAV9-Control
AAV9-mCOL1A2-PDGFRα
PDGFRα COL1A2-EGFP VEGF-C
200 μm

Figure S7 PDGF-C promotes meningeal lymphatic recovery and alleviates neuroinflammation after traumatic brain injury. Related to Figure 6. (A-E) Flow cytometry histograms and quantitative analysis of changes in brain myeloid cells for TBI + Vehicle and TBI+PDGF-C groups. (F, G) Representative images and quantification of activated microglia ($CD68^{+}IBA\text{-}1^{+}$) in the cingulum (n = 5). (H) Quantification of microglia in the cortex, dentate gyrus of hippocampus and striatum in sham, TBI + Vehicle, and TBI + PDGF-C groups (n = 5). (I) Immunofluorescence images and quantification showing that subdural PDGF-C injection does not alter cortical microglia or $CD68^{+}$ microglia in healthy mice (n = 4). (J) Immunofluorescence images and quantification showing that subdural PDGF-C injection does not alter GFAP coverage (n = 4).

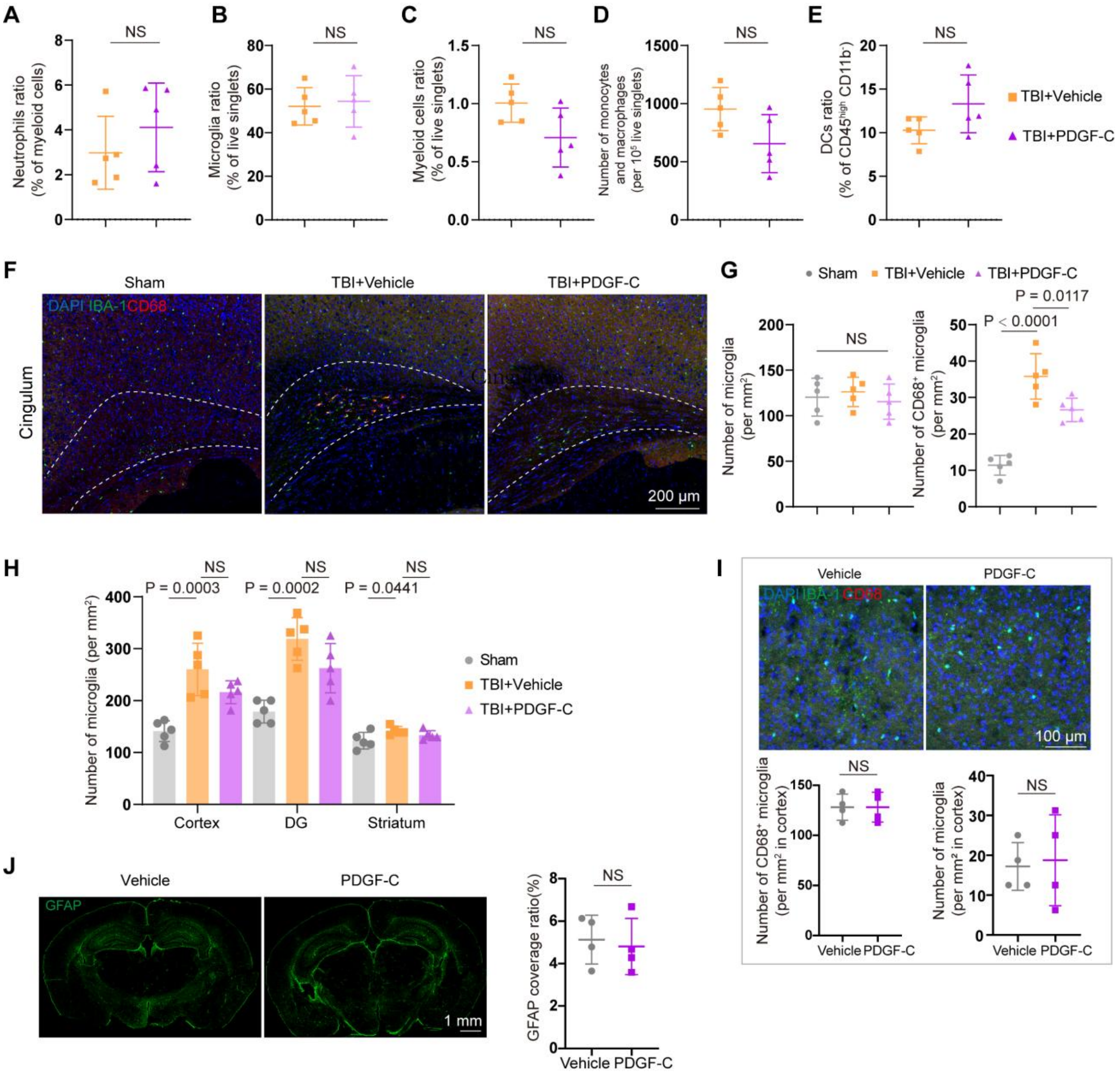

A
B
C
D
E
NS
Neutrophils ratio (% of myeloid cells)
Microglia ratio (% of live singlets)
Myeloid cells ratio (% of live singlets)
Number of monocytes and macrophages (per 10^5 live singlets)
DCs ratio (% of CD45^high CD11b^-)
TBI+Vehicle
TBI+PDGF-C
F
Sham
TBI+Vehicle
TBI+PDGF-C
DAPI IBA-1 CD68
Cingulum
200 μm
G
Sham
Number of microglia (per mm²)
Number of CD68⁺ microglia (per mm²)
P = 0.0117
P < 0.0001
H
Number of microglia (per mm²)
P = 0.0003
P = 0.0002
P = 0.0441
Cortex
DG
Striatum
I
Vehicle
PDGF-C
100 μm
Number of CD68⁺ microglia (per mm² in cortex)
Number of microglia (per mm² in cortex)
J
GFAP
1 mm
GFAP coverage ratio(%)

Table S1. Gene sets from GO functional categories used for AUCell analysis

Functional gene sets	Genes
GO: lymphangiogenesis	Vegfc, Ccbe1, Epha2, Foxc1, Ngp, Vegfa
GO: wound healing	Adipor2, Alox5, Cxcr4, Foxc2, Gata2, Hpse, Kdr, Ndnf, Serpine1, Slc12a2, Smoc2, Tafa5, Tnf, Vegfa, Vegfb, Xbp1
GO: growth factor production	Abl1, Abl2, Adtrp, Aebp1, Agt, Angptl7, Antxr1, Axin2, Bmp2, Carmil2, Cflar, Chadl, Clasp1, Clasp2, Col6a1, Colgalt1, Cpb2, Cst3, Cyp2j6, Dag1, Ddr1, Ddr2, Dpp4, Efemp2, Emilin1, Ets1, Fap, Fgfr4, Fscn1, Has2, Ier3ip1, Il6, Itgb3, Lama1, Lama2, Lamb1, Lamb2, Lamc1, Lemd3, Mad2l2, Meltf, Nid1, Notch1, Pdpn, Phldb1, Phldb2, Pparg, Prdm5, Rb1, Reck, Rgcc, Ric1, Sema5a, Slc2a10, Smad3, Smad4, Sox9, Tcf15, Tgfb1, Tgfb2, Tgfbr1, Tgfbr3, Tie1, Tnfrsf1a, Tnfrsf1b, Tnxb, Zfp469
GO: fibroblast proliferation	Abcc9, Abl1, Ager, Agt, Agtr2, Anxa2, Aqp1, B4galt7, BC004004, Bax, Bmi1, Bmyc, Brk1, Brpf1, Btc, C1ql4, Cav1, Ccna2, Ccnb1, Cd248, Cd300a, Cd74, Cdc6, Cdc73, Cdk1, Cdk4, Cdk6, Cdkn1a, Cks1b, Cks2, Col3a1, Creb1, Cripto, Ctc1, Dab2ip, Dach1, Dazap1, Ddr2, Dhx9, Dicer1, Dph1, E2f1, E2f8, Ecd, Ednra, Egf, Egfr, Emd, Ereg, Esr1, Fam114a1, Fbln1, Fbrs, Fbxo4, Fgf10, Fn1, Fndc3b, Fntb, Fosl2, Fth1, Gas6, Gng2, Gpx1, Grk2, Gstp1, Hmga2, Hras, Icmt, Ifi30, Ifng, Igf1, Il13, Inca1, Ing5, Itgb3, Jun, Kat2a, Kcnj8, Kcnn4, Kdm8, Kmt2a, Kmt2c, Lif, Lig4, Lta, Lzts2, Med25, Med31, Men1, Meox2, Mif, Mir744, Mmp9, Morc3, Morf4l1, Myb, Myc, Nbn, Ndufs4, Nf1, Ngfr, Nlrc3, Nras, Nupr1, Parp10, Pawr, Pdgfa, Pdgfb, Pdgfc, Pdgfd, Pdgfra, Pdgfrb, Pes1, Pex2, Phip, Pla2g1b, Pmaip1, Pml, Pparg, Prdx1, Prkdc, Ptges3, Ptprv, Ptprz1, Rasa1, Rasgrf1, Rnaseh2b, Rpl29, Rrn3, Serpine1, Sfrp1, Sirt6, Ski, Smarca2, Socs1, Sod2, Sp2, Sphk1, Tgfb1, Tgif1, Trim32, Trp53, Trp53inp1, Tsc2, Uts2, Uts2r, Vegfd, Wnt1, Wnt2, Wnt5a, Xrcc4, Zfp469, Zmiz1, Zmpste24
GO: fibroblast migration	2610005L07Rik, Acta2, Actr3, Adipor2, Ager, Akap12, Akt1, Apc, Appl1, Appl2, Aqp1, Arhgap4, Arid5b, Bag4, Braf, Ccn3, Cd248, Clasp2, Cln3, Coro1c, Cripto, Cygb, Ddr2, Dmtn, Fam114a1, Fer, Fgf2, Fgfr1, Fndc3b, Fut8, Gna12, Gna13, Has1, Hyal2, Ilk, Iqgap1, Itga11, Itgb1, Itgb1bp1, Itgb3, Lamtor2, Macir, Mmp1a, Mta2, Nherf1, Pak1, Pak3, Pdgfb, Pdlim1, Pip5k1a, Plec, Pml, Pmp22, Prkce, Prr5l, Ptk2, Rac1, Rcc2, Rffl, Schip1, Sdc4, Sgpl1, Slc8a1, Spag6l, Tfap2a, Tgfb1, Thbs1, Tmem201, Tns1, Tsc2, Uts2, Wdpcp, Zeb2, Zfand5, Zfp640
GO: ecm organization	Aif1, Ccm2l, Vegfc,Cd59a, Fgfr1, Fgfr4, Heg1,Ptgs2, Rgcc, Rock2, Stat5b, Wnt11

Table S2. The primer sequences used

Primer	Sequence
Vegfc-F	5' TGTGGGGAAGGAGTTTGGAG 3'
Vegfc-R	5' CAGTTACGGTCTGTGTCCAGTGTAG 3'
Gapdh-F	5' CCTTCCGTGTTCCTACCCC 3'
Gapdh-R	5' GATGCTGGTGCTGAGTATGRCG 3'
